# Associations between lifestyle, health, and clinical characteristics and circulating oxysterols and cholesterol precursors in women diagnosed with breast cancer: a cross-sectional study

**DOI:** 10.1038/s41598-024-55316-x

**Published:** 2024-02-29

**Authors:** Nina Sophia Decker, Theron Johnson, Charlotte Le Cornet, Sabine Behrens, Nadia Obi, Rudolf Kaaks, Jenny Chang-Claude, Renée Turzanski Fortner

**Affiliations:** 1 Division of Cancer Epidemiology, German Cancer Research Center (DKFZ), Heidelberg, Germany; 2 Medical Faculty Heidelberg, Heidelberg University, Heidelberg, Germany; 3 Institute for Medical Biometry and Epidemiology, University Medical Center Hamburg-Eppendorf, Hamburg, Germany; 4 Institute for Occupational and Maritime Medicine Hamburg, University Medical Center Hamburg-Eppendorf, Hamburg, Germany; 5 University Cancer Center Hamburg, Medical Center Hamburg-Eppendorf, Hamburg, Germany; 6 Department of Research, Cancer Registry of Norway, Norwegian Institute of Public Health, Oslo, Norway

**Keywords:** Breast cancer, Prognostic markers, Epidemiology

## Abstract

Despite increasing evidence that cholesterol precursors and oxysterols, oxidized cholesterol metabolites, play a role in numerous pathological processes and diseases including breast cancer, little is known about correlates of these sterols in women with breast cancer. In this study, 2282 women with breast cancer and blood draw post diagnosis were included and cross-sectional associations between circulating levels of 15 sterols/oxysterols and (a) lifestyle, anthropometric, reproductive characteristics, (b) comorbidities and medication use, and (c) breast cancer tumor and treatment characteristics were calculated using generalized linear models. Obesity was strongly associated with circulating levels of 7-dehydrocholesterol (DC) (body mass index ≥ 30 vs. 18.5–24.9 kg/m^2^: 51.7% difference) and 7-ketocholesterol (KC) (40.0% difference). After adjustment for BMI, comorbidities such as cardiovascular disease were associated with higher levels of 7-DC (26.1% difference) and lower levels of desmosterol (− 16.4% difference). Breast cancer tumor characteristics including hormone receptor status, tumor stage, and endocrine therapy were associated with lanosterol, 24-DHLan, 7b-HC, and THC (e.g., THC; tumor stage IIIa vs. I: 36.9% difference). Weaker associations were observed for lifestyle characteristics and for any of the other oxysterols. The findings of this study suggest that cholesterol precursors are strongly associated with metabolic factors, while oxysterols are associated with breast cancer tumor characteristics, warranting further investigation into the role of cholesterol precursors and oxysterols in women with breast cancer and other populations.

## Introduction

Oxysterols, oxidized metabolites of cholesterol, are produced as intermediates in normal metabolic processes such as the bile acid pathway or sex steroid synthesis^1–3^. Accumulating evidence suggests that cholesterol precursors and oxysterols may play a role in various pathological processes and diseases, including metabolic disorders^4^ and breast cancer development and progression^5,6^.

In previous studies we and others observed that circulating oxysterols 27-hydroxycholesterol (HC) and 25-HC, exhibiting estrogen receptor (ER) modulating activities, were associated with breast cancer risk^7^ and prognosis^8,9^*,* while six oxysterols including 24S-HC, 7-ketocholesterol (KC), 5a,6a-epoxycholesterol (EC), 5b6b-EC, and lanosterol were associated with cardiovascular disease mortality in women with a breast cancer diagnosis^10^. Other studies reported associations between tissue protein and mRNA expression of enzymes, such as CYP27A1, involved in oxysterol metabolism and breast cancer outcomes^11–13^.

To our knowledge, only one previous study including 1036 healthy women aged 35–65 years evaluated associations between lifestyle, dietary, reproductive, and anthropometric factors and circulating 27-HC^14^; they observed significant associations with these factors, however, changes in concentration between extreme categories were below 10%. More evidence is available on chronic and inflammatory diseases which revealed associations between cholesterol precursors and oxysterols and diabetes, atherosclerosis, and non-alcoholic fatty liver disease^4,15^. In breast cancer patients, only one smaller study (n = 58) on associations between clinical characteristics and six oxysterols was published so far, reporting lower levels of 27-HC, 7a-HC, 5b6b-EC, and THC in patients with better clinical characteristics such as smaller tumor size^9^.

Despite the increasing evidence on the role of oxysterols in breast cancer^9,13^, little is known about correlates of oxysterol concentrations in women diagnosed with breast cancer. In this large and well-characterized cross-sectional study, we aimed to identify correlates of circulating oxysterols by evaluating associations between a panel of 15 cholesterol precursors and cholesterol metabolites (both are termed “oxysterols” here) with (a) lifestyle, anthropometric, and reproductive characteristics, (b) comorbidities and medication use, (c) breast cancer tumor and treatment characteristics in a large cohort of women diagnosed with breast cancer.

## Methods

### Study population

We used data from the Mammary Carcinoma Risk Factor Investigation (MARIE) population-based patient cohort, which includes, in total, 3813 women with a histologically confirmed invasive (stage I-IV) or in situ breast cancer and aged 50–74 years at diagnosis. Enrollment took place between 2002 and 2005 in two study regions in Germany (Hamburg and Rhine-Neckar-Karlsruhe). Blood samples were drawn at recruitment, and information on anthropometrics, lifestyle, reproduction, comorbidities, and medication use at baseline were obtained from participants via personal interviews at baseline. In telephone interviews in 2009 (follow-up 1) and in 2015 (follow-up 2) updated information on lifestyle, comorbidities, and endocrine therapy (exogenous selective estrogen receptor modulators (SERMs) such as tamoxifen, aromatase inhibitors (AIs)) was retrieved for the preceding time interval. If self-reported information on endocrine therapy was not available, data from medical records was used. Participants were asked at baseline to indicate comorbidities of a listed set of diseases with the option to state other diseases as free text. An adapted version of the Charlson Comorbidity Index (CCI)^20^ comprises the following comorbidities: myocardial infarction, circulatory disorders in the legs (arterial occlusive disease), stroke, dementia, chronic lung disease (asthma, bronchitis), collagenosis, gastric ulcer, chronic liver disease (hepatitis, liver cirrhosis), diabetes, chronic bladder or kidney disease, congestive heart failure, and hemiplegia. The variable cardiovascular disease (CVD) includes participants who reported at least one of the following: angina pectoris, circulatory disorders in the legs, heart attack, hypertension, or stroke, as well as participants who reported CVD before the respective follow-up. Data on cholesterol and total energy intake prior to diagnosis was obtained via self-administered, validated food frequency questionnaires^21^, similar to the food frequency questionnaire from the European Prospective Investigation into Cancer and Nutrition (EPIC). In the current study, 2282 participants with stage I to stage IIIa breast cancers and available baseline blood samples were included. Sample selection of this study has been reported previously^8^; median time between diagnosis and blood collection was 3.7 months (range, —14.5 months to 57.6 months), with eight participants having blood drawn median 5 months prior to diagnosis due to original recruitment as control (Table S1).

Ethical approval for the MARIE study was obtained from the ethics committees of the Heidelberg University, the Hamburg Medical Council, and the Medical Board of the State of Rhineland-Palatine, and ethical approval for this study was obtained from the ethics committees of the Heidelberg University and the United States Department of Defense Human Research Protections Office. The study was conducted in accordance with the Declaration of Helsinki. All study participants provided written informed consent.

### Laboratory

In total, 5 cholesterol precursors and 10 oxysterols were measured: lanosterol (lan), 24,25-dihydrolanosterol (24-DHLan), desmosterol (desmos), 7-dehydrocholesterol (7-DC), 24,25-epoxycholesterol (24,25-EC), 27-hydroxycholesterol (27-HC) (systematic name: (25R), 26-hydroxycholesterol), 25-hydroxycholesterol (25-HC), 24S–hydroxycholesterol (24S-HC), 22R–hydroxycholesterol (22R-HC), 5α,6α-epoxycholesterol (5a,6a-EC), 5β,6β-epoxycholesterol (5b,6b-EC), 5α,6β-dihydroxycholestanol (other name: 3b,5a,6b-Cholestanetriol; THC), 7α-hydroxycholesterol (7a-HC), 7β-hydroxycholesterol (7b-HC), 7-ketocholesterol (7-KC). For the ease of reading, the term “oxysterols” is used for both, cholesterol precursors (lanosterol, 24-DHLan, desmosterol, 7-DC, 24,25-EC) and cholesterol metabolites (27-HC, 25-HC, 5a,6a-EC, 5b,6b-EC, 7a-HC, 7b-HC, 7-KC, THC) in the following text. Oxysterol levels were quantified using a mass spectrometer with electrospray ionization by Biocrates LifeSciences (Innsbruck, Austria). Inter-assay coefficients of variation (CV) were assessed by including 16 blinded replicate quality controls. Analyte concentrations and CVs are included in the supplements (Table S2). In brief, majority of oxysterols had mean intra-assay CVs below 20%, and mean inter-assay CVs below 25%, except of 5a6a-EC (31.8%), 24-DHLan (33.7%), 7-KC (42.4%), 7b-HC (74.9%). It is worth noting that high CVs are observed in oxysterols with very low concentrations, and concentrations of the quality control samples were generally lower than those in the study samples. We excluded 22R-HC and 24,25-EC from the main analyses due to the high number of values below LOD (> 88%). Estradiol concentrations were quantified using an ELISA^8^, and 25-hydroxyvitamin D [25(OH)D] was quantified previously using the OCTEIA 25(OH)D enzyme immunoassay^22,23^.

### Oxysterol classification

**Figure 1:**
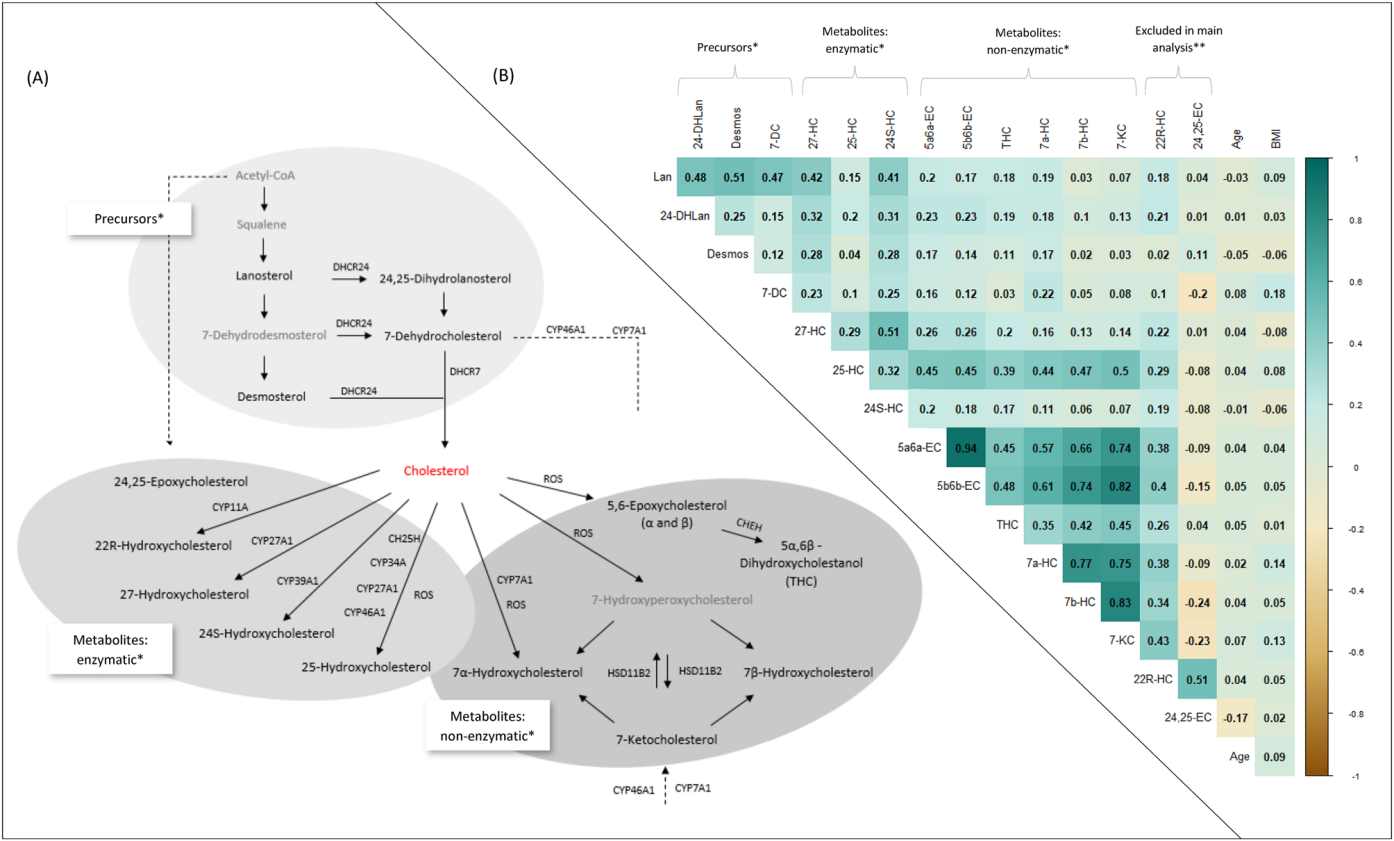
Metabolic pathways and correlations of oxysterols. (A) Metabolic pathways of oxysterols. Metabolic pathways of oxysterols and corresponding enzymes or reactive oxygen species (ROS). Grey color indicates oxysterols that were not measured in our study (needed for completeness of this figure). *Please note that these are not fixed subgroups, but created with the purpose of providing a better overview of the results. Adapted from^1,16–19^ (**B)** Correlation matrix of circulating oxysterols: MARIE patient cohort. Spearman partial correlation coefficients, adjusted for age and center; using imputed, log_2_-transformed values. n = 2269, except 7-DC (n = 2209), 24-DHLan (n = 1019), 22R-HC (n = 263), 24,25-EC (n = 86). Abbreviations: 24S-HC = 24S-hydroxycholesterol; 7a-HC = 7α-hydroxycholesterol; 7b-HC = 7β-hydroxycholesterol; ; 7-KC = 7-ketocholesterol; 5a6a-EC = 5α,6α-epoxycholesterol; 5b6b-EC = 5β,6β-epoxycholesterol; THC = 5α,6β-dihydroxycholestanol; 7-DC = 7-dehydrocholesterol; Lan = lanosterol; 24-DHLan = 24,25-dihydrolanosterol; Desmos = desmosterol; 22R-HC = 22R- hydroxycholesterol; 24,25-EC = 24,25-epoxycholesterol; BMI = body mass index. *Please not that these are not fixed subgroups, but created with the purpose of providing a better overview of the results. **22R-HC and 24,25-EC are excluded from cross-sectional analyses due to high amount of values below LOD. Adapted from^10^.

Correlations within this panel of circulating oxysterols have been described previously^10^, and are presented in Fig. 1b. In brief, strong correlations were observed between metabolites 7a-HC, 7b-HC, 7-KC, 5a6a-EC, and 5b6b-EC (0.57 ≥ r ≥ 0.94). THC and 25-HC were moderately correlated with these oxysterols (THC: 0.35 ≥ r ≥ 0.48; 25-HC: 0.45 ≥ r ≥ 0.50). Moderate correlations were observed between 27-HC and 24S-HC (r = 0.51), and between lanosterol and desmosterol (r = 0.51), 24-DHLan (r = 0.48), and 7-DC (r = 0.47). Correlations between remaining oxysterols were weaker (r < │0.42│).

Based on these correlations and the reported metabolic pathways of the oxysterols (enzymatic or non-enzymatic conversion) (Fig. 1A)^1,16–19^, we created the following groups of oxysterols with the purpose of providing a better overview of the results, but we recognize that these are not fixed subgroups:

- Cholesterol-precursors: lanosterol, desmosterol, 7-DC, 24-DHLan
- Cholesterol-metabolites, enzymatic conversion: 27-HC, 25-HC , 24S-HC
- Cholesterol-metabolites, non-enzymatic conversion: 5a6a-EC, 5b6b-EC, THC, 7a-HC, 7b-HC, 7-KC

Further information on metabolic pathways of these oxysterols is provided in the supplements.

### Statistics

We applied a log_2_-transformation to all biomarker concentrations to obtain approximately normal distributions. Values below the limit of detection (LOD) were observed for the following biomarkers: desmosterol, n = 11; 7-DC, n = 150; 25-HC, n = 89; 24S-HC, n = 7; 7b-HC, n = 14; THC, n = 492. Apart from THC, values below LOD were imputed using half of the detection limit stratified by study region; additionally, we conducted sensitivity analyses without imputation. Values exceeding the calibration range were detected for the following biomarkers and excluded from all analyses: 25-HC, n = 89; 24S-HC, n = 2; 5a6a-EC, n = 1; 5b6b-EC, n = 4; 7a-HC, n = 11; 7b-HC, n = 10; 25-HC, n = 89. The outliers for the following oxysterols were detected using the generalized ESD many-outlier procedure^24^: lanosterol, n = 1; desmosterol, n = 30; 24-DHLan, n = 1; 27-HC, n = 1; 25-HC, n = 15; 24S-HC, n = 8; 5a6a-EC, n = 10; 5b6b-EC, n = 4; 7a-HC, n = 1; 7b-HC, n = 13; 7-KC, n = 8. Due to the relatively high number of outliers for desmosterol, we conducted sensitivity analyses excluding these outliers.

Exposures significantly associated with at least one oxysterol and with a difference of > 10% between exposure categories are listed in Tables 1, 2, 3, whereas exposures with weaker associations (≤ 10% difference) are listed in Supplemental Data (Tables S3-S5). If two variables were highly related, we included the representative variable in the main table (e.g. BMI), and the others in the Supplemental Data (e.g. waist-hip-ratio, WHR).

**Table 1:** Cross-sectional associations between selected anthropometric, lifestyle, and reproductive characteristics and oxysterol concentrations by metabolic pathway.

Exposure	Categories	n (%)^a^	Geometric mean (95% CI), nM	% diff	Geometric mean (95% CI), nM	% diff	Geometric mean (95% CI), nM	% diff	Geometric mean (95% CI), nM	% diff
Cholesterol precursors^b^		2282 (100%)	Lanosterol (n = 2282)		24-DHLan (n = 1027)		Desmosterol (n = 2281)		7-DC (n = 2221)	
Age at diagnosis (years)	< 55	332 (14.5)	539.8 (512.8,568.3)	Ref	39.2 (36.1,42.6)	Ref	1774.3 (1680.3,1873.5)	Ref	470.0 (417.2,529.4)	Ref
	55—59	452 (19.8)	544.1 (520.6,568.5)	0.79	40.5 (37.5,43.8)	3.32	1797.8 (1716.1,1883.4)	1.33	436.4 (394.1,483.2)	− 7.14
	60—64	675 (29.6)	546.3 (526.9,566.3)	1.20	40.1 (37.8,42.7)	2.34	1726.2 (1661.7,1793.2)	2.71	519.8 (478.0,565.3)	10.61
	65—69	566 (24.8)	531.3 (510.8,552.6)	− 1.58	38.4 (35.8,41.1)	− 2.22	1621.5 (1555.5,1690.3)	− 8.61	538.6 (491.3,590.5)	14.61
	70 +	257 (11.3)	485.1 (457.5,514.3)	− 10.14	41.4 (37.2,46.1)	5.52	1607.0 (1510.6,1709.5)	− 9.43	493.2 (430.5,565.0)	4.94
*p*-value, extreme categories				0.017		0.394		0.012		0.322
BMI (kg/m^2^)^c^	< 18.5	35 (1.5)	520.3 (444.1,609.5)	− 0.94	43.3 (34.1,55.1)	8.70	1787.7 (1512.8,2112.6)	1.66	456.9 (316.1,660.3)	6.02
	18.5—24.9	1031 (45.2)	525.2 (510.1,540.8)	Ref	39.9 (38.0,41.8)	Ref	1758.5 (1705.1,1813.5)	Ref	430.9 (402.8,461.0)	Ref
	25—29.9	848 (37.2)	532.4 (515.4,549.9)	1.37	38.6 (36.5,40.9)	− 3.03	1689.3 (1632.6,1748.0)	− 3.93	526.4 (488.2,567.5)	22.16
	≥ 30	368 (16.1)	566.2 (539.1,594.6)	7.80	41.9 (38.4,45.7)	5.10	1599.4 (1518.8,1684.3)	− 9.04	653.8 (582.6,733.8)	51.73
*p*-value, extreme categories				0.010		0.333		0.002		< .0001
Parity^d^	0	382 (16.7)	535.3 (510.3,561.6)	Ref	38.9 (35.9,42.2)	Ref	1787.9 (1699.6,1880.7)	Ref	447.1 (400.0,499.8)	Ref
	1	643 (28.2)	548.5 (528.6,569.2)	2.45	39.9 (37.5,42.5)	2.62	1705.9 (1640.5,1773.9)	− 4.59	527.1 (483.7,574.4)	17.90
	2 +	1257 (55.1)	526.3 (512.4,540.5)	− 1.70	39.9 (38.1,41.8)	2.55	1682.4 (1635.7,1730.3)	− 5.90	496.3 (466.6,528.0)	11.01
*p*-value, extreme categories				0.542		0.599		0.040		0.109
Education	Low	1356 (59.4)	526.8 (513.4,540.6)	Ref	39.8 (38.0,41.7)	Ref	1672.1 (1627.0,1718.4)	Ref	494.9 (465.9,525.7)	Ref
	Medium	598 (26.2)	548.0 (527.3,569.5)	4.02	40.1 (37.6,42.8)	0.82	1764.5 (1694.2,1837.6)	5.52	506.0 (462.8,553.2)	2.24
	High	328 (14.4)	538.2 (510.6,567.2)	2.45	38.8 (35.6,42.3)	− 2.46	1744.3 (1650.1,1843.8)	4.32	482.2 (426.9,544.6)	− 2.57
*p*-value, extreme categories				0.480		0.621		0.184		0.709

Cholesterol metabolites: “enzymatic pathway”^b^		2282 (100%)	27-HC (n = 2282)		25-HC (n = 2282)		24S-HC (n = 2279)			
Age at diagnosis (years)	< 55	332 (14.5)	204.0 (198.7,209.5)	Ref	19.7 (18.5,21.1)	Ref	93.3 (90.1,96.7)	Ref		
	55—59	452 (19.8)	209.7 (205.0,214.5)	2.81	20.4 (19.3,21.6)	3.58	93.6 (90.8,96.5)	0.30		
	60—64	675 (29.6)	210.4 (206.5,214.3)	3.14	19.7 (18.8,20.6)	− 0.15	96.6 (94.3,99.1)	3.56		
	65—69	566 (24.8)	211.0 (206.8,215.4)	3.46	19.9 (18.9,20.9)	0.72	93.2 (90.7,95.8)	− 0.14		
	70 +	257 (11.3)	209.1 (202.9,215.5)	2.51	19.5 (18.1,21.1)	− 0.95	94.1 (90.4,98.0)	0.84		
*p*-value, extreme categories				0.310		0.994		0.846		
BMI (kg/m^2^)^c^	< 18.5	35 (1.5)	218.3 (201.3,236.8)	2.13	19.4 (15.9,23.7)	− 1.59	100.7 (90.3,112.3)	4.93		
	18.5—24.9	1031 (45.2)	213.8 (210.6,217.0)	Ref	19.7 (19.0,20.5)	Ref	96.0 (94.1,97.9)	Ref		
	25—29.9	848 (37.2)	206.1 (202.7,209.5)	− 3.60	19.5 (18.7,20.3)	− 1.37	93.8 (91.7,95.9)	− 2.32		
	≥ 30	368 (16.1)	204.0 (198.9,209.2)	− 4.58	21.4 (20.1,22.8)	8.65	91.1 (88.1,94.2)	− 5.08		
*p*-value, extreme categories				0.002		0.025		0.010		
Parity^d^	0	382 (16.7)	208.6 (203.5,213.8)	Ref	20.2 (19.0,21.5)	Ref	93.2 (90.1,96.3)	Ref		
	1	643 (28.2)	212.1 (208.1,216.2)	1.68	20.0 (19.1,21.0)	− 0.91	94.9 (92.5,97.4)	1.91		
	2 +	1257 (55.1)	208.2 (205.3,211.0)	− 0.19	19.7 (19.0,20.4)	− 2.61	94.5 (92.8,96.3)	1.48		
*p*-value, extreme categories				0.894		0.458		0.448		
Education	Low	1356 (59.4)	207.8 (205.0,210.6)	Ref	19.7 (19.1,20.3)	Ref	93.4 (91.7,95.1)	Ref		
	Medium	598 (26.2)	213.2 (209.0,217.5)	2.61	20.5 (19.6,21.6)	4.28	97.3 (94.8,99.9)	4.20		
	High	328 (14.4)	208.6 (203.1,214.4)	0.41	19.5 (18.2,20.8)	− 1.21	93.3 (90.0,96.8)	− 0.09		
*p*-value, extreme categories				0.793		0.750		0.965		

Cholesterol metabolites: “non-enzymatic pathway”^b^		2282 (100%)	5a6a-EC (n = 2281)		5b6b-EC (n = 2278)		THC (n = 2282)			
Age at diagnosis (years)	< 55	332 (14.5)	26.9 (25.3,28.6)	Ref	105.4 (99.1,112.0)	Ref	3.9 (3.4,4.5)	Ref		
	55—59	452 (19.8)	27.9 (26.5,29.4)	3.85	109.8 (104.1,115.7)	4.20	4.6 (4.1,5.2)	16.01		
	60—64	675 (29.6)	28.0 (26.8,29.2)	4.17	111.1 (106.4,116.0)	5.44	4.8 (4.4,5.3)	22.27		
	65—69	566 (24.8)	28.6 (27.3,30.0)	6.36	113.0 (107.8,118.4)	7.21	4.8 (4.4,5.4)	22.77		
	70 +	257 (11.3)	28.0 (26.1,30.0)	4.07	111.5 (103.9,119.5)	5.8	4.7 (4.0,5.5)	18.97		
*p*-value, extreme categories				0.343		0.196		0.083		
BMI (kg/m^2^)^c^	< 18.5	35 (1.5)	29.1 (24.1,35.2)	5.85	122.6 (101.5,148.0)	13.98	5.3 (3.5,8.2)	17.86		
	18.5—24.9	1031 (45.2)	27.5 (26.6,28.5)	Ref	107.5 (103.9,111.3)	Ref	4.5 (4.2,4.9)	Ref		
	25—29.9	848 (37.2)	27.8 (26.7,28.9)	1.01	110.0 (105.8,114.3)	2.26	4.5 (4.1,4.9)	− 1.18		
	≥ 30	368 (16.1)	29.6 (27.9,31.4)	7.77	119.3 (112.5,126.5)	10.97	5.3 (4.6,6.0)	17.03		
*p*-value, extreme categories				0.033		0.003		0.046		
Parity^d^	0	382 (16.7)	27.2 (25.7,28.9)	Ref	108.7 (102.7,115.1)	Ref	4.4 (3.9,5.1)	Ref		
	1	643 (28.2)	28.3 (27.1,29.6)	3.94	111.6 (106.7,116.6)	2.60	4.9 (4.4,5.4)	10.87		
	2 +	1257 (55.1)	28.0 (27.1,28.9)	2.78	110.5 (107.0,114.0)	1.60	4.6 (4.2,4.9)	2.54		
Education *p*-value, extreme categories				0.419		0.635		0.742		
	Low	1356 (59.4)	27.8 (26.9,28.7)	Ref	111.0 (107.6,114.4)	Ref	4.6 (4.3,4.9)	Ref		
	Medium	598 (26.2)	29.3 (27.9,30.6)	5.26	113.5 (108.4,118.9)	2.30	5.1 (4.6,5.7)	10.70		
	High	328 (14.4)	26.3 (24.7,28.1)	− 5.21	103.1 (96.9,109.8)	− 7.05	4.0 (3.4,4.6)	− 14.24		
*p*-value, extreme categories				0.141		0.043		0.060		

Cholesterol metabolites: “non-enzymatic pathway”^**b**^		2282 (100%)	7a-HC (n = 2271)		7b-HC (n = 2272)		7-KC (n = 2282)			
Age at diagnosis (years)	< 55	332 (14.5)	273.5 (257.2,290.8)	Ref	170.5 (154.4,188.3)	Ref	181.6 (165.9,198.7)	Ref		
	55—59	452 (19.8)	293.4 (278.3,309.2)	7.27	185.3 (170.2,201.8)	8.68	213.3 (197.5,230.4)	17.49		
	60—64	675 (29.6)	293.9 (281.5,306.8)	7.45	191.3 (178.5,205.1)	12.22	205.0 (192.5,218.4)	12.91		
	65—69	566 (24.8)	297.4 (283.8,311.7)	8.75	195.3 (181.0,210.7)	14.53	212.9 (198.7,228.1)	17.23		
	70 +	257 (11.3)	269.3 (251.1,288.7)	− 1.55	187.7 (167.7,210.1)	10.09	215.7 (194.6,239.0)	18.79		
*p*-value, extreme categories				0.940		0.160		0.005		
BMI (kg/m^2^)^c^	< 18.5	35 (1.5)	299.4 (247.3,362.4)	11.53	229.0 (168.2,311.8)	31.53	233.6 (177.1,308.1)	26.01		
	18.5—24.9	1031 (45.2)	268.4 (259.2,277.9)	Ref	174.1 (164.6,184.2)	Ref	185.4 (176.1,195.1)	Ref		
	25—29.9	848 (37.2)	298.4 (287.1,310.1)	11.16	192.0 (180.4,204.3)	10.27	211.0 (199.4,223.3)	13.86		
	≥ 30	368 (16.1)	328.5 (309.8,348.3)	22.37	214.8 (195.3,236.1)	23.36	259.4 (238.1,282.7)	39.97		
*p*-value, extreme categories				< .0001		0.0002		< .0001		
Parity^d^	0	382 (16.7)	287.7 (271.7,304.7)	Ref	185.4 (169.0,203.4)	Ref	203.0 (186.6,220.9)	Ref		
	1	643 (28.2)	291.3 (278.7,304.5)	1.26	191.0 (177.8,205.2)	3.04	208.2 (195.1,222.2)	2.57		
	2 +	1257 (55.1)	287.8 (278.8,297.1)	0.03	186.4 (177.1,196.2)	0.57	206.0 (196.6,215.8)	1.45		
*p*-value, extreme categories				0.994		0.917		0.771		
Education	Low	1356 (59.4)	290.9 (282.1,300.0)	Ref	191.3 (182.0,201.1)	Ref	205.6 (196.5,215.1)	Ref		
	Medium	598 (26.2)	286.5 (273.6,300.0)	− 1.52	186.3 (173.0,200.7)	− 2.59	214.9 (200.9,229.9)	4.53		
	High	328 (14.4)	284.0 (266.7,302.4)	− 2.37	174.7 (157.9,193.4)	− 8.65	192.7 (175.8,211.4)	− 6.24		
*p*-value, extreme categories				0.505		0.120		0.223		

Geometric means (95% CI) calculated using generalized linear models adjusted for age at diagnosis (continuous; except exposure category age), BMI (continuous; except exposure category BMI), and study region (Hamburg, Rhine-Neckar-Karlsruhe; except exposure category study region). Table 1 includes exposures significantly associated with at least one oxysterol and with > 10% difference between exposure categories.

Significance level after Bonferroni correction *p* < 0.0001.

^a^missing values due to values below LOD or values exceeding calibration range.

^b^please note that these are not fixed subgroups but only created for a better overview.

^c^BMI = body mass index, < 18.5 = underweight, 18.5–24.9 = normal weight, 25–29.9 = overweight, 30 +  = obese.

^d^parity = number of full-term births.

nM = nanomolar; LOD = level of detection; p-diff = percentage difference between extreme categories; BMI = body mass index; lan = lanosterol; 24-DHLan = 24,25-dihydrolanosterol; desmos = desmosterol; 7-DC = 7-dehydrocholesterol; 27-HC = 27-hydroxycholesterol; 25-HC = 25-hydroxycholesterol; 24S-HC = 24S-hydroxycholesterol; 5a6a-EC = 5α,6α-epoxycholesterol; 5b6b-EC = 5β,6β-epoxycholesterol; THC = 5α,6β-dihydroxycholestanol; 7a-HC = 7α-hydroxycholesterol; 7b-HC = 7β-hydroxycholesterol; 7-KC = 7-ketocholesterol.

**Table 2:** Cross-sectional associations between selected comorbidities and medication use at baseline and oxysterol concentrations by metabolic pathway.

Exposure	Categories	n (%)^c^	Geometric mean (95% CI), nM	% diff	Geometric mean (95% CI), nM	% diff	Geometric mean (95% CI), nM	% diff	Geometric mean (95% CI), nM	% diff	
Cholesterol precursors		2282 (100%)	Lanosterol (n = 2282)		24-DHLan (n = 1027)		Desmosterol (n = 2281)		7-DC (n = 2221)		
CVD	No	1136 (49.8)	543.5 (528.1,559.3)	. Ref	40.6 (38.7,42.6)	Ref	1866.9 (1811.8,1923.6)	Ref	442.6 (414.5,472.6)	Ref	
	Yes	1146 (50.2)	524.7 (509.9,539.9)	− 3.45	38.8 (36.9,40.8)	− 4.57	1561.0 (1515.2,1608.2)	− 16.38	558.2 (522.1,596.9)	26.13	
*p*-value, extreme categories				0.099		0.201		< .0001		< .0001	
Diabetes	No	2070 (90.7)	540.1 (529.1,551.3)	Ref	95.5 (94.2,96.9)	Ref	1738.1 (1700.9,1776.2)	Ref	491.5 (468.5,515.6)	Ref	
	Yes	207 (9.1)	475.7 (445.2,508.3)	− 11.93	84.2 (80.5,88.1)	− 11.84	1427.2 (1331.1,1530.2)	− 17.89	528.5 (452.4,617.3)	7.52	
*p*-value, extreme categories				0.0004		< .0001		< .0001		0.384	
Statin use	No	1378 (60.4)	555.8 (542.0,570.0)	Ref	40.0 (38.4,41.8)	Ref	1786.6 (1739.8,1834.8)	Ref	516.9 (487.3,548.2)	Ref	
	Yes	300 (13.1)	450.7 (427.2,475.6)	− 18.91	34.6 (31.3,38.3)	− 13.53	1468.5 (1387.6,1554.2)	− 17.80	470.8 (414.8,534.4)	− 8.91	
	Unknown	601 (26.3)	530.6 (510.9,551.1)	− 4.53	41.4 (38.9,44.2)	3.52	1658.4 (1593.2,1726.3)	− 7.18	462.7 (423.3,505.6)	− 10.49	
*p*-value, extreme categories				< .0001		0.010		< .0001		0.191	
Aspirin use	No	1416 (62.1)	542.1 (528.7,555.8)	. Ref	39.4 (37.7,41.1)	Ref	1758.3 (1712.6,1805.3)	Ref	505.0 (476.4,535.2)	Ref	
	Yes	265 (11.6)	499.1 (471.1,528.9)	− 7.93	37.7 (34.0,41.9)	− 4.22	1551.2 (1459.4,1648.7)	− 11.78	527.7 (461.0,604.1)	4.51	
	Unknown	601 (26.3)	530.8 (510.9,551.5)	− 2.08	41.4 (38.8,44.2)	5.27	1658.5 (1593.0,1726.6)	− 5.68	463.0 (423.7,506.0)	− 8.30	
*p*-value, extreme categories				0.010		0.454		0.0002		0.557	

Cholesterol metabolites: “enzymatic pathway”^b^			27-HC (n = 2282)		25-HC (n = 2282)		24S-HC (n = 2279)				
CVD	No	1136 (49.8)	211.2 (208.1,214.3)	Ref	19.9 (19.2,20.6)	Ref	95.5 (93.6,97.4)	Ref			
	Yes	1146 (50.2)	207.5 (204.5,210.6)	− 1.72	19.9 (19.2,20.6)	0.14	93.3 (91.5,95.2)	− 2.27			
*p*-value, extreme categories				0.113		0.959		0.118			
Diabetes	No	2070 (90.7)	210.9 (208.7,213.2)	Ref	19.8 (19.3,20.3)	Ref	95.5 (94.2,96.9)	Ref			
	Yes	207 (9.1)	194.0 (187.5,200.8)	− 8.00	20.7 (19.0,22.5)	4.54	84.2 (80.5,88.1)	− 11.84			
*p*-value, extreme categories				< .0001		0.325		< .0001			
Statin use	No	1378 (60.4)	207.9 (205.2,210.6)	Ref	19.6 (19.0,20.3)	Ref	93.8 (92.1,95.4)	Ref			
	Yes	300 (13.1)	209.0 (203.2,214.9)	0.54	20.5 (19.2,22.0)	4.77	97.3 (93.8,101.0)	3.80			
	Unknown	601 (26.3)	213.0 (208.8,217.2)	2.44	20.2 (19.2,21.2)	2.85	94.4 (92.0,97.0)	0.69			
*p*-value, extreme categories				0.734		0.228		0.077			
Aspirin use	No	1416 (62.1)	209.5 (206.8,212.2)	Ref	19.9 (19.3,20.5)	Ref	95.1 (93.4,96.7)	Ref			
	Yes	265 (11.6)	200.6 (194.7,206.7)	− 4.24	19.2 (17.8,20.7)	− 3.43	90.9 (87.4,94.7)	− 4.34			
	Unknown	601 (26.3)	212.9 (208.8,217.1)	1.63	20.2 (19.2,21.2)	1.40	94.4 (91.9,96.9)	− 0.71			
*p*-value, extreme categories				0.009		0.394		0.046			

Cholesterol metabolites: “non-enzymatic pathway”^b^		2282 (100%)	5a6a-EC (n = 2281)		5b6b-EC (n = 2278)		THC (n = 2282)				
CVD	No	1136 (49.8)	28.3 (27.3,29.3)	Ref	111.6 (107.8,115.5)	Ref	4.8 (4.5,5.2)	Ref			
	Yes	1146 (50.2)	27.6 (26.7,28.6)	− 2.44	109.4 (105.7,113.2)	1.72	4.4 (4.1,4.8)	− 7.77			
*p*-value, extreme categories				0.336		0.430		0.162			
Diabetes	No	2070 (90.7)	28.1 (27.4,28.8)	Ref	111.0 (108.3,113.7)	Ref	4.7 (4.4,5.0)	Ref			
	Yes	207 (9.1)	26.5 (24.5,28.8)	− 5.52	105.3 (97.3,114.0)	− 5.10	4.2 (3.5,5.0)	− 11.34			
*p*-value, extreme categories				0.186		0.217		0.212			
Statin use	No	1378 (60.4)	27.9 (27.1,28.8)	Ref	109.5 (106.2,112.8)	Ref	4.6 (4.3,4.9)	Ref			
	Yes	300 (13.1)	27.7 (26.0,29.6)	− 0.64	111.0 (104.1,118.5)	1.46	4.3 (3.7,5.0)	− 5.98			
	Unknown	601 (26.3)	28.2 (26.9,29.5)	0.89	112.5 (107.5,117.7)	2.77	4.9 (4.4,5.5)	7.16			
*p*-value, extreme categories				0.861		0.692		0.456			
Aspirin use	No	1416 (62.1)	28.1 (27.3,29.0)	Ref	110.8 (107.6,114.2)	Ref	4.7 (4.4,5.0)	Ref			
	Yes	265 (11.6)	26.5 (24.8,28.5)	− 5.68	104.2 (97.3,111.7)	− 5.97	3.8 (3.3,4.5)	− 18.17			
	Unknown	601 (26.3)	28.2 (26.9,29.5)	0.03	112.4 (107.4,117.7)	1.45	4.9 (4.4,5.5)	5.08			
*p*-value, extreme categories				0.132		0.110		0.022			

Cholesterol metabolites: “non-enzymatic pathway”^b^		2282 (100%)	7a-HC (n = 2271)		7b-HC (n = 2272)		7-KC (n = 2282)				
CVD	No	1136 (49.8)	289.5 (279.7,299.6)	Ref	187.2 (177.1,197.9)	Ref	203.5 (193.5,214.1)	Ref			
	Yes	1146 (50.2)	288.1 (278.4,298.1)	− 0.49	187.9 (177.8,198.5)	0.36	208.7 (198.5,219.4)	2.53			
*p*-value, extreme categories				0.847		0.930		0.504			
Diabetes	No	2070 (90.7)	286.8 (279.8,293.9)	Ref	187.7 (180.3,195.3)	Ref	205.3 (198.0,212.9)	Ref			
	Yes	207 (9.1)	308.8 (285.3,334.2)	7.67	185.1 (162.8,210.3)	− 1.37	213.1 (189.7,239.4)	3.80			
*p*-value, extreme categories				0.081		0.840		0.549			
Statin use	No	1378 (60.4)	284.0 (275.5,292.7)	Ref	182.7 (173.9,191.8)	Ref	202.0 (193.2,211.2)	Ref			
	Yes	300 (13.1)	297.7 (279.1,317.5)	4.82	188.5 (169.8,209.2)	3.18	218.0 (198.2,239.7)	7.91			
	Unknown	601 (26.3)	295.2 (282.0,309.0)	3.95	198.4 (184.3,213.6)	8.59	209.5 (195.9,224.1)	3.73			
*p*-value, extreme categories				0.196		0.594		0.155			
Aspirin use	No	1416 (62.1)	287.6 (279.2,296.4)	Ref	184.9 (176.2,194.0)	Ref	207.8 (198.9,217.1)	Ref			
	Yes	265 (11.6)	280.5 (261.8,300.5)	− 2.49	177.9 (159.2,198.9)	− 3.76	190.2 (171.8,210.6)	− 8.45			
	Unknown	601 (26.3)	295.1 (281.9,308.9)	2.61	198.3 (184.2,213.5)	7.27	209.4 (195.8,223.9)	0.77			
*p*-value, extreme categories				0.512		0.537		0.118			

Geometric means (95% CI) calculated using generalized linear models adjusted for age at diagnosis (continuous; except exposure category age), BMI (continuous; except exposure category BMI), and study region (Hamburg, Rhine-Neckar-Karlsruhe; except exposure category study region). Table 2 includes exposures significantly associated with at least one oxysterol and with > 10% difference between exposure categories.

Significance level after Bonferroni correction *p* < 0.0001.

^a^missing oxysterol values due to levels below LOD or levels exceeding calibration range; missing exposure categories values: diabetes, n = 5; statin use, n = 3.

^b^please note that these are not fixed subgroups but only created for a better overview,

nM = nanomolar; LOD = level of detection; p-diff = percentage difference between extreme categories; CVD = cardiovascular disease; 24-DHLan = 24,25-dihydrolanosterol; 7-DC = 7-dehydrocholesterol; 27-HC = 27-hydroxycholesterol; 25-HC = 25-hydroxycholesterol; 24S-HC = 24S-hydroxycholesterol; 5a6a-EC = 5α,6α-epoxycholesterol; 5b6b-EC = 5β,6β-epoxycholesterol; THC = 5α,6β-dihydroxycholestanol; 7a-HC = 7α-hydroxycholesterol; 7b-HC = 7β-hydroxycholesterol; 7-KC = 7-ketocholesterol.

**Table 3:** Cross-sectional associations between selected breast cancer tumor and treatment characteristics and oxysterol concentrations by metabolic pathway.

Exposure	Categories	n (%)^a^	Geometric mean (95% CI), nM	% diff	Geometric mean (95% CI), nM	% diff	Geometric mean (95% CI), nM	% diff	Geometric mean (95% CI), nM	% diff	
Cholesterol precursors^b^		2282 (100%)	Lanosterol (n = 2282)		24-DHLan (n = 1027)		Desmosterol (n = 2281)		7-DC (n = 2221)		
ER/PR *p*-value, extreme categories	ER/PR +	1583 (69.4)	530.7 (518.3,543.4)	Ref	39.6 (38.1,41.3)	Ref	1717.4 (1675.1,1760.8)	Ref	512.1 (484.7,541.0)	Ref	
	ER + /PR- or ER-/PR +	376 (16.5)	521.9 (497.3,547.6)	− 1.67	38.7 (35.5,42.1)	− 2.49	1673.6 (1590.3,1761.3)	− 2.55	462.9 (413.9,517.6)	− 9.62	
	ER/PR-	323 (14.2)	565.4 (536.7,595.6)	6.54	41.3 (38.0,45.0)	4.25	1692.1 (1601.5,1787.8)	− 1.48	460.5 (408.1,519.6)	− 10.08	
				0.030		0.385		0.629		0.116	
Stage	I	1137 (49.8)	514.1 (500.0,528.6)	Ref	38.3 (36.5,40.2)	Ref	1762.4 (1711.4,1814.9)	Ref	498.8 (467.7,531.9)	Ref	
	IIa	718 (31.5)	546.6 (527.8,566.0)	6.31	39.8 (37.5,42.3)	3.88	1661.3 (1601.1,1723.8)	− 5.74	466.2 (429.5,506.0)	− 6.54	
	IIb	273 (12.0)	553.5 (523.1,585.7)	7.67	40.8 (37.2,44.8)	6.50	1575.7 (1484.4,1672.7)	− 10.59	559.3 (489.9,638.4)	12.13	
	IIIa	154 (6.7)	595.4 (552.3,642.0)	15.82	49.3 (43.3,56.1)	28.54	1755.4 (1621.2,1900.6)	− 0.40	511.1 (429.2,608.7)	2.48	
*p*-value, extreme categories				0.0003		0.0004		0.926		0.797	
Endocrine therapy^c^	None	351 (15.4)	559.6 (532.3,588.3)	Ref	42.3 (39.0,45.9)	Ref	1670.8 (1584.7,1761.6)	Ref	460.4 (409.9,517.2)	Ref	
	Tam + AI	742 (32.5)	542.4 (524.0,561.4)	− 3.08	39.7 (37.5,42.0)	− 6.18	1710.8 (1649.6,1774.3)	2.39	533.0 (491.7,577.7)	15.75	
	Tam only	855 (37.5)	511.6 (495.4,528.2)	− 8.58	37.0 (34.9,39.2)	− 12.59	1734.2 (1676.4,1794.0)	2.39	487.8 (452.8,525.4)	5.94	
	AI only	255 (11.2)	541.7 (510.5,574.9)	− 3.19	44.6 (40.2,49.5)	5.36	1605.8 (1508.1,1709.8)	− 3.89	451.9 (393.2,519.3)	− 1.86	
	Unknown	79 (3.5)	574.9 (517.6,638.6)	2.74	42.4 (35.3,51.0)	0.24	1852.5 (1657.8,2070.1)	10.88	565.2 (444.0,719.3)	22.74	
*p*-value, extreme categories				0.312		0.205		0.469		0.042	

Cholesterol metabolites: “enzymatic pathway”^b^		2282 (100%)	27-HC (n = 2282)		25-HC (n = 2282)		24S-HC (n = 2279)				
ER/PR	ER/PR +	1583 (69.4)	208.5 (206.0,211.1)	Ref	20.0 (19.4,20.6)	Ref	94.2 (92.6,95.7)	Ref			
	ER + /PR- or ER-/PR +	376 (16.5)	209.1 (204.0,214.4)	0.29	19.1 (18.0,20.3)	− 4.69	93.9 (90.8,97.1)	− 0.26			
	ER/PR-	323 (14.2)	213.8 (208.1,219.6)	2.53	20.1 (18.8,21.5)	0.52	96.2 (92.8,99.7)	2.16			
*p*-value, extreme categories				0.096		0.889		0.287			
Stage	I	1137 (49.8)	208.2 (205.2,211.2)	Ref	19.4 (18.8,20.1)	Ref	93.1 (91.4,95.0)	Ref			
	IIa	718 (31.5)	209.8 (206.1,213.6)	0.79	20.1 (19.2,21.0)	3.44	95.2 (92.9,97.5)	2.18			
	IIb	273 (12.0)	211.4 (205.3,217.7)	1.54	20.2 (18.8,21.7)	4.15	94.6 (91.0,98.4)	1.59			
	IIIa	154 (6.7)	212.1 (204.0,220.5)	1.86	21.7 (19.7,23.8)	11.51	100.0 (95.0,105.4)	7.40			
*p*-value, extreme categories				0.383		0.036		0.012			
Endocrine therapy^c^	None	351 (15.4)	213.9 (208.5,219.5)	Ref	20.3 (19.0,21.6)	Ref	96.5 (93.2,99.8)	Ref			
	Tam + AI	742 (32.5)	210.1 (206.4,213.9)	− 1.76	19.9 (19.1,20.8)	− 1.77	95.4 (93.2,97.7)	− 1.07			
	Tam only	855 (37.5)	206.1 (202.8,209.6)	− 3.62	19.8 (19.0,20.6)	− 2.38	92.5 (90.5,94.6)	− 4.08			
	AI only	255 (11.2)	207.8 (201.5,214.2)	− 2.86	19.3 (17.9,20.8)	− 4.85	93.9 (90.1,97.8)	− 2.65			
	Unknown	79 (3.5)	222.7 (211.0,235.1)	4.11	19.9 (17.5,22.8)	− 1.72	98.6 (91.7,106.1)	2.27			
*p*-value, extreme categories				0.265		0.649		0.613			

Cholesterol metabolites: “enzymatic pathway”^b^		2282 (100%)	5a6a-EC (n = 2281)		5b6b-EC (n = 2278)		THC (n = 2282)				
ER/PR	ER/PR +	1583 (69.4)	28.3 (27.5,29.2)	Ref	112.3 (109.2,115.5)	Ref	4.5 (4.3,4.8)	Ref			
	ER + /PR- or ER-/PR +	376 (16.5)	27.6 (26.0,29.3)	− 2.59	109.4 (103.3,115.9)	− 2.56	4.5 (4.0,5.1)	− 0.38			
	ER/PR-	323 (14.2)	26.6 (24.9,28.3)	− 6.28	103.0 (96.8,109.6)	− 8.30	5.3 (4.6,6.1)	17.22			
*p*-value, extreme categories				0.434		0.013		0.045			
Stage	I	1137 (49.8)	27.6 (26.7,28.6)	Ref	109.4 (105.8,113.1)	Ref	4.1 (3.8,4.5)	Ref			
	IIa	718 (31.5)	27.9 (26.8,29.1)	1.07	110.3 (105.8,115.0)	0.89	5.0 (4.5,5.5)	19.88			
	IIb	273 (12.0)	28.4 (26.5,30.4)	2.76	110.3 (103.1,118.0)	0.87	5.5 (4.7,6.4)	32.04			
	IIIa	154 (6.7)	30.1 (27.5,32.9)	8.9	120.2 (109.9,131.6)	9.97	5.7 (4.6,7.0)	36.89			
*p*-value, extreme categories				0.085		0.053		0.005			
Endocrine therapy^c^	None	351 (15.4)	28.0 (26.4,29.7)	Ref	108.0 (101.8,114.7)	Ref	5.3 (4.7,6.1)	Ref			
	Tam + AI	742 (32.5)	28.6 (27.4,29.8)	2.14	112.8 (108.2,117.5)	4.36	4.4 (4.0,4.8)	− 18.25			
	Tam only	855 (37.5)	27.6 (26.6,28.7)	− 1.34	110.6 (106.4,114.9)	2.36	4.5 (4.1,4.9)	− 16.06			
	AI only	255 (11.2)	27.1 (25.2,29.1)	− 3.2	107.0 (99.7,114.9)	− 0.97	4.9 (4.2,5.8)	− 7.95			
	Unknown	79 (3.5)	28.4 (25.0,32.2)	1.33	109.9 (97.0,124.6)	1.74	5.3 (4.0,7.0)	− 0.67			
*p*-value, extreme categories				0.571		0.249		0.016			

Cholesterol metabolites: “enzymatic pathway”^d^		2282 (100%)	7a-HC (n = 2271)		7b-HC (n = 2272)		7-KC (n = 2282)				
ER/PR	ER/PR +	1583 (69.4)	294.7 (286.5,303.1)	Ref	191.6 (183.1,200.6)	Ref	211.1 (202.5,220.0)	Ref			
	ER + /PR- or ER-/PR +	376 (16.5)	290.3 (274.1,307.5)	− 1.50	197.7 (180.2,216.9)	3.18	210.0 (193.0,228.5)	− 0.51			
	ER/PR-	323 (14.2)	259.7 (244.1,276.3)	− 11.88	158.5 (143.4,175.2)	− 17.30	179.4 (163.8,196.6)	− 14.98			
*p*-value, extreme categories				0.0003		0.0007		0.003			
Stage	I	1137 (49.8)	292.7 (283.1,302.6)	Ref	188.6 (178.7,199.0)	Ref	205.9 (196.0,216.2)	Ref			
	IIa	718 (31.5)	287.4 (275.6,299.6)	− 1.82	190.9 (178.4,204.3)	1.24	203.0 (190.9,215.9)	− 1.40			
	IIb	273 (12.0)	282.4 (263.9,302.2)	− 3.52	171.0 (153.2,190.8)	− 9.32	207.2 (187.6,228.8)	0.63			
	IIIa	154 (6.7)	277.9 (254.0,304.1)	− 5.05	194.8 (168.5,225.3)	3.31	221.0 (193.6,252.2)	7.33			
*p*-value, extreme categories				0.290		0.681		0.326			
Endocrine therapy^c^	None	351 (15.4)	266.4 (250.9,282.8)	Ref	163.6 (148.5,180.3)	Ref	198.1 (181.5,216.3)	Ref			
	Tam + AI	742 (32.5)	299.4 (287.4,311.9)	12.38	193.0 (180.6,206.3)	17.9	217.1 (204.3,230.6)	9.56			
	Tam only	855 (37.5)	299.5 (288.3,311.2)	12.44	200.2 (188.2,213.0)	22.36	207.5 (196.2,219.6)	4.76			
	AI only	255 (11.2)	263.2 (245.2,282.5)	− 1.93	167.4 (149.3,187.8)	2.32	190.1 (171.3,211.0)	− 4.03			
	Unknown	79 (3.5)	261.2 (230.6,296.0)	− 1.93	179.3 (146.5,219.5)	9.59	180.3 (149.9,216.8)	− 8.99			
*p*-value, extreme categories				0.002		0.006		0.093			

Geometric means (95% CI) calculated using generalized linear models adjusted for age at diagnosis (continuous; except exposure category age), BMI (continuous; except exposure category BMI), and study region (Hamburg, Rhine-Neckar-Karlsruhe; except exposure category study region). Table 3 includes exposures significantly associated with at least one oxysterol and with > 10% difference between exposure categories.

Significance level after Bonferroni correction *p* < 0.0001.

^a^missing oxysterol values due to levels below LOD or levels exceeding calibration range; missing exposure categories values: grade, n = 11.

^b^Please note that these are not fixed subgroups but only created for a better overview.

^c^Endocrine therapy: None = neither tamoxifen nor AI; Tam + AI = tamoxifen and aromatase inhibitors; Tam only = tamoxifen (no aromatase inhibitors); AI only = aromatase inhibitors (no tamoxifen).

nM = nanomolar; LOD = level of detection; p-diff = percentage difference between extreme categories; tam = tamoxifen; AI = aromatase inhibitors; 24-DHLan = 24,25-dihydrolanosterol; 7-DC = 7-dehydrocholesterol; 27-HC = 27-hydroxycholesterol; 25-HC = 25-hydroxycholesterol; 24S-HC = 24S-hydroxycholesterol; 5a6a-EC = 5α,6α-epoxycholesterol; 5b6b-EC = 5β,6β-epoxycholesterol; THC = 5α,6β-dihydroxycholestanol; 7a-HC = 7α-hydroxycholesterol; 7b-HC = 7β-hydroxycholesterol; 7-KC = 7-ketocholesterol.

We used generalized linear models to evaluate cross-sectional associations between log_2_-transformed oxysterol concentrations and case-related characteristics including (a) lifestyle, anthropometric, and reproductive characteristics, (b) comorbidities and medication use at baseline, (c) tumor and breast cancer treatment characteristics. After back-transformation, geometric means of oxysterol by exposure category were obtained, percentage difference (% difference) of the geometric means between the categories, and the related *p*-values were calculated. All models were adjusted for age at diagnosis, BMI, and study region. In sensitivity analyses, we analyzed all exposures strongly associated with oxysterol concentrations in the primary analysis (% difference > 10%) together in a single model (Table S6). To discriminate endocrine therapy use at the time of blood collection, we investigated associations between endocrine therapy and oxysterols among participants with blood collection ≥ 3 months after their diagnosis (Table S5).

Proportion of variance explained by the case-related characteristics (R^2^) was calculated using a linear regression model.

All statistical tests were two-tailed and *p* < 0.05 was considered significant. Statistical analyses were conducted using SAS 9.4 (SAS Institute Inc., Cary, NC, USA) and R (version 4.2.1).

## Results

The median age of study participants at diagnosis was 63 years (range: 50–75 years). A total of 45.2% were normal weight (BMI 18.5–24.9 km/m^2^), 37.2% were overweight (BMI 25–29.9 km/m^2^), and 16.1% were obese (BMI ≥ 30 km/m^2^). The majority of participants (91%) was postmenopausal at the time of diagnosis, while the remaining participants had uncertain menopausal status due to hysterectomy or menopausal hormone therapy. Most participates had ER- and PR-positive tumors (69.4%), 16.5% had ER-positive/PR-negative or ER-negative/PR-positive tumors, and 14.2% had ER- and PR-negative tumors. The majority of participates reported endocrine therapy use (32.5% tamoxifen and AI use, 37.5% tamoxifen use, 11.2% AI use), and 15.4% of participants reported never endocrine therapy use.

Of the 2282 participants included in this study, complete observations were available for lanosterol, 27-HC, 25-HC, THC, and 7-KC. Sample size was smaller due to values below LOD or above calibration range for desmosterol (n = 2281), 5a6a-EC (n = 2281), 5b6b-EC (n = 2278), 7b-HC (n = 2272), 7a-HC (n = 2271), 7-DC (n = 2221), and 24-DHLan (n = 1027).

**Figure 2:**
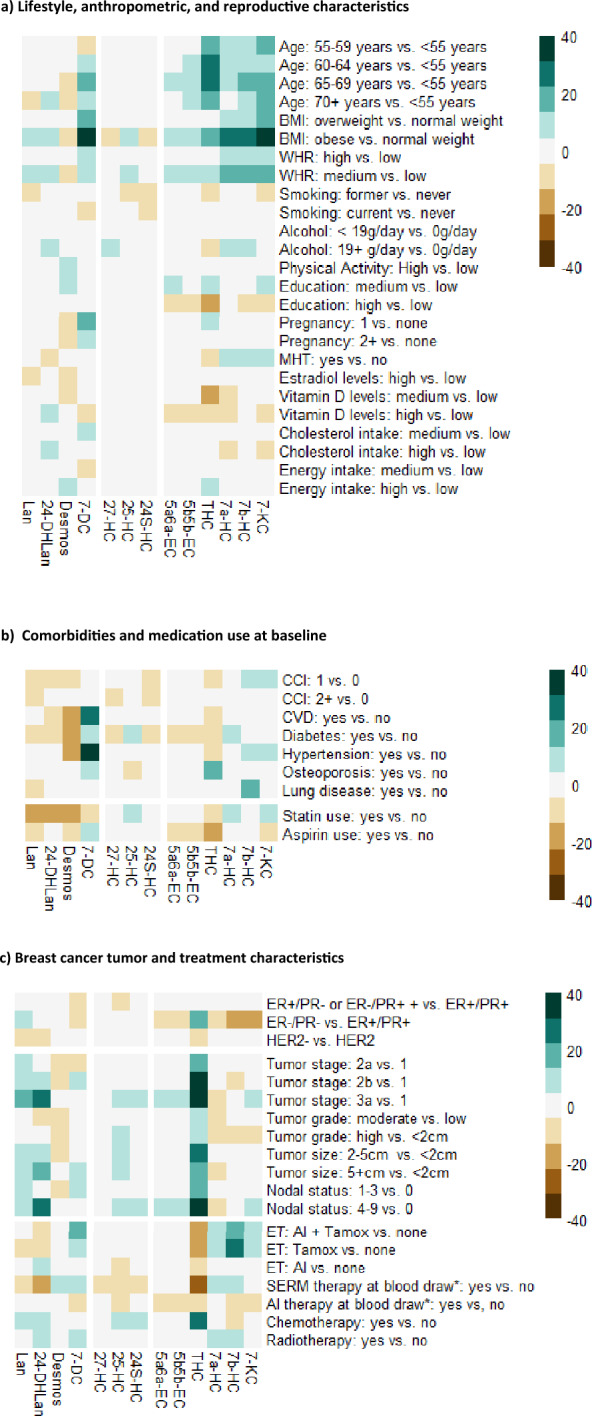
Heatmap illustrating cross-sectional associations between oxysterol concentrations and (**a**) lifestyle, anthropometric, and reproductive characteristics, (**b**) comorbidities and medication use, (**c**) breast cancer tumor and treatment characteristics. Associations are shown using percentage difference of oxysterol geometric mean concentrations (adjusted for age at diagnosis (except exposure category age), BMI (except exposure category BMI), and study region (Hamburg, Rhine-Neckar-Karlsruhe; except exposure category study region). BMI = body mass index; WHR = waist-hip-ration; MHT = menopausal hormone therapy; CCI = Charlson Comorbidity Index; CVD = cardiovascular disease; ER/PR = estrogen-receptor / progesterone receptor; ET = endocrine therapy; SERM = selective estrogen receptor modulator such as tamoxifen; AI = aromatase inhibitor. *endocrine therapy use among women whose blood was drawn ≥ 3 months after diagnosis. Abbreviations: lan = lanosterol; 24-DHLan = 24,25-dihydrolanosterol; desmos = desmosterol; 7-DC = 7-dehydrocholesterol; 27-HC = 27-hydroxycholesterol; 25-HC = 25-hydroxycholesterol; 24S-HC = 24S-hydroxycholesterol; 5a6a-EC = 5α,6α-epoxycholesterol; 5b6b-EC = 5β,6β-epoxycholesterol; THC = 5α,6β-dihydroxycholestanol; 7a-HC = 7α-hydroxycholesterol; 7b-HC = 7β-hydroxycholesterol; 7-KC = 7-ketocholesterol. Missing oxysterol values due to levels below LOD or levels exceeding calibration range. Missing exposure categories values: hypertension, n = 3; diabetes, n = 5; statin use, n = 3; grade, n = 11; tumor size, n = 2.

Figure 2 displays cross-sectional associations between oxysterol concentrations and (a) lifestyle, anthropometric, and reproductive characteristics, (b) comorbidities and medication use, (c) breast cancer tumor and treatment characteristics. Tables 1, 2, 3 contain the variables showing a difference of more than 10% between extreme categories (represented by darker color in the heatmaps). The remaining variables are included in the Supplemental Data. The significance level after adjusting for multiple comparisons using the Bonferroni correction is 0.0001 (13 metabolites and 14 case characteristics, 8 comorbidities and medication use characteristics, and 10 breast cancer characteristics).

### (a) Lifestyle, anthropometric, and reproductive characteristics

The most consistent associations were observed for circulating oxysterols and BMI. High BMI was associated with almost all investigated oxysterols, though the magnitude and direction of association differed (Table 1). Obesity (BMI ≥ 30 kg/m^2^) versus normal BMI (BMI 18.5–24.9 kg/m^2^) was associated with higher levels of 7-DC, THC, 7a-HC, 7b-HC, and 7-KC (range: 17.0% (THC) to 51.7% (7-DC) difference). Weak positive associations were observed for lanosterol, 25-HC, 5a6a-EC, 5b6b-EC (≤ 11% difference, BMI ≥ 30 vs. 18.5–24.9 kg/m^2^), and weak inverse associations were observed for desmosterol, 27-HC, and 24S-HC (≤ −9.0% difference, BMI ≥ 30 vs. 18.5–24.9 kg/m^2^). These associations were confirmed in further analyses using waist-hip-ratio (WHR), another measure of body composition, showing similar effects to associations with BMI; after adjustment for BMI, results between WHR and oxysterols were attenuated (Table S3).

Older age was associated with higher levels of THC and 7-KC (e.g., 7-KC, 18.8% difference, age ≥ 70 vs. < 55 years), whereas associations with other oxysterols were weaker (< 15% difference).

Parity was associated with higher levels of 7-DC (17.9% difference, 1 vs. 0 full-term pregnancies), but weakly with the other oxysterols (< 12% difference 1 or 2 + vs. 0 full-term pregnancies) (Table 1). Menopausal hormone therapy (yes vs. no) and circulating estradiol levels (≥ 0.08 nM vs. < 0.08 nM) were weakly associated with circulating oxysterols (< 10% difference) (Table S3).

Nutritional factors including estimated total cholesterol intake (upper vs. lower tertile, in g/day), total energy intake (upper vs. lower tertile, in kcal/day), and circulating vitamin D concentrations (upper vs. lower tertile, in nmol/L), were weakly associated with circulating oxysterol levels (< 6% difference, < 7% difference, < 12% difference, respectively) (Table S3).

Weak associations were observed between circulating oxysterol concentrations and level of education (high vs. low, < 15% difference), smoking status (current vs. never, < 13% difference), alcohol consumption (19 + g/day vs. 0 g/day, < 10% difference), and leisure time physical activity (≥ 28 vs. < 28 met*h/week < 11% difference) (Table S3).

### (b) Comorbidities and medication use at baseline

Metabolic diseases at baseline were strongly associated with circulating levels of desmosterol and 7-DC. Women who reported CVD had lower levels of desmosterol (− 16.4% difference) and higher levels of 7-DC (26.1% difference, Table 2), and women with diabetes had lower levels of desmosterol (− 17.9% difference, Table 2). Similar associations were observed among women with hypertension who had higher levels of desmosterol and 7-DC (− 19.6% difference and 31.8% difference, respectively, Table S4.

Statin use was associated with lower levels of lanosterol (− 18.9% difference), desmosterol (− 17.8% difference, and 24-DHLan (− 13.5% difference). Aspirin use was associated with lower levels of THC (− 18.2% difference) (Table 2).

Osteoporosis was associated with higher levels of THC (19.9% difference), and chronic lung diseases was associated with higher levels of 7b-HC (16.3%) (Table S4).

Other oxysterols were weakly associated with comorbidities or medication use at recruitment (≤ 12% difference) (Table S4).

### (c) Breast cancer tumor and treatment characteristics

Participants with hormone receptor negative tumors had lower concentrations of 7a-HC (− 11.9% difference), 7b-HC (− 17.3% difference), and 7-KC (− 15.0% difference), and higher levels of THC (17.2% difference) as compared to hormone receptor positive tumors (Table 3). HER2-status (negative vs. positive) was not associated with oxysterol levels (Table S5).

Higher tumor stage (stage IIIa vs. I; Table 3) was associated with higher levels of lanosterol, 24-DHLan, and THC (between 15.8% and 36.9% difference). This is in line with findings for tumor size and nodal status, both included in the category staging system: larger tumor size (≥ 5cm vs. < 5 cm) was associated with higher levels of lanosterol, 24-DHLan, and THC (between 12.1% and 20.6% difference), and higher number of positive lymph nodes (4–9 vs. 0) was associated with higher levels of 24-DHLan and THC (25.8% and 35.6% difference, respectively). Weaker associations were observed for tumor grade (≤ 12% difference) (Table S5).

Women reporting ever tamoxifen or ever tamoxifen and AI use, had lower levels of THC (− 16.1% and − 18.3% difference, respectively), and higher levels of 7b-HC (22.4% and 17.9% difference, respectively) as compared to women reporting never endocrine therapy use. Among women reporting tamoxifen and AI use, slightly higher levels of 7-DC (15.8% difference) were observed as compared to those reporting never endocrine therapy use. The associations with remaining oxysterols and women who reported tamoxifen or tamoxifen and AI were weaker (< 14% difference), as well as the associations between any oxysterols and women who reported AI use only (ever AI vs. never endocrine therapy, < 8% difference; Table 3).

Women reporting ever chemotherapy use had higher levels of THC (29.2% difference) as compared to those not reporting chemotherapy use, whereas weaker associations (< 11% difference) were observed for other oxysterols with chemotherapy (ever vs. never chemotherapy) and radiotherapy (ever vs. never radiotherapy; Table S5).

### Sensitivity analyses

Excluding outliers for desmosterol (n = 30) attenuated some of the results minimally (e.g., age: − 9.4% to − 7.4% difference; CVD: − 16.4% to − 15.3% difference) (data not shown), however, significant results remained significant and interpretation of the results did not change. Excluding outliers for the other oxysterols had minimal impact on the results (< 10% difference).

In secondary models including all exposures strongly associated (> 10% difference and *p* < 0.05) with oxysterol concentrations in the primary analysis (e.g. 7-DC: parity, CVD, tumor stage, endocrine therapy use) in addition to age, BMI, and study region, most of the associations were attenuated (e.g. 7-DC: change from 51.7% to 38.4% difference, BMI ≥ 30 vs. 18.5–24.9 kg/m^2^), while some associations yielded stronger results (e.g. THC: change from 17.03% to 19.8% difference, BMI ≥ 30 vs. 18.5–24.9 kg/m^2^) (Table S6); overall, magnitude and direction of effects were similar to the results from the main analysis.

Associations between endocrine therapy use and oxysterols among participants with blood collection ≥ 3 months after diagnosis had similar direction of effects to the ever endocrine therapy use, including strong inverse associations between tamoxifen + AI use and THC and strong positive associations between tamoxifen use/ tamoxifen + AI use and 7a-HC and 7b-HC, while associations between tamoxifen use and lanosterol and 24-DHLan yielded stronger results (e.g. lanosterol: change from − 8.58% to − 16.92% difference; 24-DHLan: change from − 12.59 to − 22.25% difference, tamoxifen use vs. no use) (Table S5).

### Variability

The factors associated with circulating oxysterols explained less than 7% of variability in concentrations for each individual oxysterol. Highest proportion of explained variance (R^2^) was observed for desmosterol (6.5%) and 7-DC (4.5%) when including all of the variables significantly associated with the respective oxysterol. Hypertension and CVD were the strongest predictors, contributing 5.0% and 3.7% of observed variation in desmosterol levels, respectively, and 2.6% and 2.1%, to the observed variation in 7-DC levels, respectively.

## Discussion

The findings of this study suggest that case and tumor characteristics were associated with selected cholesterol precursors and cholesterol metabolites reflecting different pathways of cholesterol synthesis. The most consistent associations were observed for measures of obesity including BMI and WHR and circulating levels 7-DC, 7a-HC, 7b-HC, and 7-KC. Furthermore, metabolic diseases including CVD and diabetes were associated with cholesterol precursors 7-DC and desmosterol, even after mutually adjusting these variables for each other. Breast cancer tumor characteristics such as hormone-receptor status, tumor stage, and endocrine therapy were strongly associated with circulating levels of the cholesterol precursors lanosterol and 24-DHLan, and the ROS-produced oxysterols THC and 7b-HC. Weak associations with all patient and tumor characteristics were observed for the ER-modulating oxysterols 27-HC and 25-HC. Overall, the case-characteristics evaluated in this study accounted for a low proportion of the observed variance in oxysterol concentrations.

### Cholesterol precursors

The strongest association across all categories was observed for obesity, which was strongly associated with higher levels of 7-DC and 7-KC. 7-DC is a direct cholesterol precursor, while 7-KC is a secondary cholesterol metabolite that can be formed either from other cholesterol metabolites including 7a-HC and 7b-HC (strong correlations, r ≥ 0.75) or from the cholesterol precursor 7-DC (weak correlations, r < 0.1). Cholesterol precursors are considered markers for cholesterol biosynthesis^15^, and since cholesterol levels are commonly elevated in obesity, this may explain the observed associations between high BMI and circulating 7-DC. Furthermore, obesity is associated with oxidative stress^25,26^, a condition in which cholesterol oxidation and oxysterol formation was reported to be increased^27,28^. It is possible that the 7-DC/7-KC pathway may play a role in obesity through increased cholesterol synthesis or oxidative stress.

A previous study reported positive associations between BMI and cholesterol precursors (desmosterol, r = 0.418; lanosterol, r = 0.454) in healthy participants^29^, while weaker associations between BMI and cholesterol precursors were observed in patients with familial combined hyperlipidemia (desmosterol, r = 0.219; lanosterol, r = 0.203)^30^, in healthy participants (r ≤ 0.1)^31^, and in one of our previous studies (r < 0.2) evaluating continuous BMI and cholesterol precursors in women with a breast cancer diagnosis^10^. These results suggest that altered 7-DC and 7-KC levels are found only in women with obesity (BMI ≥ 30 kg/m^2^) or with potential other factors related to obesity.

Besides obesity, cholesterol precursors were strongly associated with reported disease at baseline including CVD, hypertension, and diabetes. While desmosterol, an intermediate of the Bloch-pathway, was inversely associated with diabetes and CVD, 7-DC, an intermediate of the Kandutsch-Russel-pathway, was positively associated with diabetes and CVD. A potential differential regulation of the Kandutsch-Russel (7-DC) and the Bloch (desmosterol) pathway has been suggested previously^15^, and could explain the opposite direction of effect for these cholesterol precursors. Furthermore, the use of statins, which function by blocking HMG-CoA reductase, the first enzyme of the cholesterol synthesis pathway, was associated with lower levels of lanosterol, desmosterol, and 24-DHLan but not with 7-DC, supporting the indication of a differential regulation of cholesterol biosynthesis pathways.

The role of cholesterol precursors as mediators in metabolic processes is of increasing interest. Previous human studies reported altered concentrations of intermediates of cholesterol synthesis in various metabolic and inflammatory diseases such as diabetes, atherosclerosis, and non-alcoholic fatty liver disease^4,15^. To our knowledge, we are the first to report associations between cholesterol precursors and metabolic disorders in women diagnosed with breast cancer.

A dual role of lanosterol/24-DHLan and 7-DC was also observed with respect to breast cancer characteristics. Adverse breast cancer tumor characteristics such as higher tumor stage were associated with higher levels of lanosterol and 24-DHLan and slightly lower levels of 7-DC; and endocrine therapy use was associated with lower levels of 24-DHLan and higher levels of 7-DC. Sensitivity analyses including only women whose blood was drawn three months after their diagnosis indicated that lower levels of lanosterol and 24-DHLan were indeed associated with endocrine therapy use.

To date, little is known about the role of lanosterol and 24-DHLan in breast cancer. While it has been reported that 24-DHLan may play a role in cholesterol metabolism^32^, the effect of lanosterol and 24-DHLan in breast cancer development and progression has not been evaluated. Tamoxifen has been reported to block enzymes of the cholesterol biosynthesis pathway leading to the accumulation of cholesterol precursors such as desmosterol and 7-DC^33^. In this study, we observed slightly higher levels of desmosterol and 7-DC in women reporting tamoxifen use, while significantly lower concentrations of the early cholesterol precursor lanosterol and 24-DHLan were observed, warranting further studies to investigate the underlying biological mechanisms between cholesterol precursors and breast cancer tumor and treatment characteristics.

### Cholesterol metabolites: enzymatic

We observed weak associations between 27-HC, 25-HC, 24S-HC and any of the patient and tumor characteristics evaluated. In line with the weak associations observed in this study for 27-HC, a previous study of our working group in healthy women within the EPIC-Heidelberg cohort reported significant but small changes in concentration by lifestyle, diet, reproductive or anthropometric factors (≤ │10│% difference)^14^.

Consistent with our findings, no association between cholesterol-lowering drugs and 27-HC levels was found in two larger studies of breast cancer patients and healthy postmenopausal women^14,34^, whereas in another study including 42 breast cancer patients, a decrease in 27-HC levels was observed after two weeks of statin treatment^13^.

While 27-HC, 25-HC, and 24S-HC were weakly associated with tamoxifen or AI use in this study, a clinical study including 29 breast cancer patients receiving tamoxifen (n = 15) or AI (n = 14) over a 28-day period, reported that tamoxifen treatment significantly decreased circulating levels of 24-HC and 25-HC, whereas AI treatment significantly increased 27-HC levels^35^. While Dalenc et al. investigated hormone-receptor positive invasive and metastatic breast cancer patient, we included invasive, non-metastatic women with hormone-receptor positive and negative tumors in this study. Kimbung et al. reported that patients with high tumor expression of CYP27A1, the enzyme converting cholesterol to 27-HC, were less likely to receive endocrine treatment (OR = 0.62 (0.45–0.87))^12^, however, comparison between circulating 27-HC and tissue content are difficult as associations were reported to be weak (r = 0.029)^36^.

While Kloudova et al. observed differences in 27-levels by tumor size or tumor stage, we observed weak association for 27-HC in this study, potentially due to differences in hormone-receptor status and endocrine therapy between the study populations. Consistent with the results of this cross-sectional study, a previous study of 287 breast cancer cases from the EPIC-Heidelberg cohort found no association between hormone receptor status, tumor stage, and serum 27-HC levels^37^.

### Cholesterol metabolites: non-enzymatic

Age at diagnosis was significantly associated with 7-KC but not with the other oxysterols, which is in line with a previous study on 58 women diagnosed with breast cancer^9^ reporting strong correlations between 7-KC and age (r = 0.94), but weaker correlations for other oxysterols (r ≤ │0.41).

Furthermore, obesity has been associated with higher levels of 7-KC, 7a-HC and 7b-HC, which can be interconverted. A previous study on serum levels of several oxysterols in men and women found no difference between obese (defined as BMI > 30 without dyslipidemia, diabetes or hypertension) and metabolically healthy participants, whereas participants with metabolic syndrome (defined as at least 3 of the following factors: abdominal obesity, elevated triglycerides, reduced HDL, elevated blood pressure, or elevated plasma glucose) had higher levels of 7a-HC, 7b-HC, Triol (here: THC), and 25-HC as compared to metabolically healthy controls^38^. As participants with obesity (BMI ≥ 30 kg/m^2^ or WHR ≥ 0.85) in the current study were more similar to participants with the metabolic syndrome in Tremblay-Franco’s study due to reported comorbidities such as diabetes and hypertension, our results are consistent with those previously reported for patients with metabolic syndrome.

It is possible that the elevated levels of 7-KC, 7a-HC, and 7b-HC observed in women with obesity may be due to oxidative stress associated with obesity and concomitant diseases^25,26^, as all of these oxysterols can be produced via ROS. Alternatively, 7-KC can also be formed enzymatically from the cholesterol precursor 7-DC, which was strongly associated with BMI; therefore, it is possible that higher levels of 7-KC, 7a-HC, and 7b-C may result from elevated cholesterol synthesis. The findings of this study warrant further investigation into the role of 7-KC, 7a-HC, and 7b-HC in obesity and metabolic syndrome.

Higher levels of THC were observed in hormone receptor-negative as compared to positive tumors, and correspondingly, higher levels of THC were found in women not treated with endocrine therapy and in women treated with chemotherapy, the indicated treatment for hormone receptor-negative tumors. Furthermore, THC levels were associated with unfavorable tumor characteristics such as higher tumor stage, suggesting that THC may play a role in breast cancer characteristics and the corresponding treatment. In line with our findings, a previous study reported significantly lower levels of cholestane-3β,5α,6βtriol (THC) in early breast cancer stages compared with more advanced stages (Ia vs. other)^9^.

Furthermore, participants who reported regular aspirin use had lower THC levels as compared to non-aspirin users. Aspirin, a non-steroidal anti-inflammatory drug (NSAID), reduces inflammation by binding and inhibiting pro-inflammatory cyclooxygenase (COX1 and COX2), which regulate prostaglandin production. Recent studies have shown beneficial effects of aspirin on cardiovascular health^39^ and on breast cancer development and progression^40^, however, associations between aspirin use and THC have not yet been reported.

Higher levels of 7b-HC were observed in hormone receptor-positive as compared to negative tumors and in women who reported tamoxifen therapy use, indicated for hormone-receptor positive tumors. While 7b-HC has not yet been investigated in relation to breast cancer, Kloudova et al. reported lower 7a-HC levels in participants with smaller tumors as compared to those with larger tumors^9^; due to strong correlations between 7a-HC and 7b-HC (r = 0.77), the findings of Kloudova’s study may be comparable to those of the current study.

While we observed weak associations with breast cancer tumor and treatment characteristics for the other oxysterols, Kloudova et al. found differences in 27-HC and 5b6b-EC levels by tumor size and tumor stage^9^. As 5b6b-EC is a precursor of THC, it is also possible that higher metabolism of these oxysterols may have caused altered THC levels, resulting in the strong associations for THC and weaker associations for 5a6a-EC and 5b6b-EC in this study.

### Strength and limitations

The strength of this study lies in the evaluation of a panel of oxysterols and a number of case-related characteristics including lifestyle, reproduction, comorbidities, and clinical characteristics. With 2282 participants, this is the largest study to date characterizing circulating oxysterols in a breast cancer population.

A limitation of this study is that blood cholesterol levels were not measured in this cohort, so adjustment for total cholesterol levels was not possible, and we present absolute oxysterol concentrations rather than oxysterol/cholesterol ratios. However, it should be noted that circulating total cholesterol has been shown to be weakly to moderately correlated with oxysterols and cholesterol precursors in healthy participants (0.19 ≤ r ≤ 0.53)^7,31^, in patients with familial combined hyperlipidemia (r ≤ │0.2│)^30^, and in breast cancer patients (27-HC, r = 0.384)^13^.

Changes of biomarker concentration over time could not be evaluated since blood samples were collected at only one time point. In addition, we had incomplete information on the start and end date of endocrine therapy and incomplete information on the use of medications such as statins, limiting the assessment of medication use at the time of blood collection. Blood in this cohort study was not collected fasting, however, previous studies reported weak associations between 27-HC, 25-HC, 24S-HC, and 7a-HC and fasting status (8–11 h vs. ≥ 17 h fasting: ≤ 4% difference; 12–16 h vs. > 17h fasting, ≤ 8% difference)^41^, and between 27-HC and fasting status (≥ 3 h vs. < 3 h fasting, − 2.79%)^14^.

While relatively good intra-person reproducibility of 0.62 to 0.91 over a 1-year period was demonstrated for most oxysterols, intra-person reproducibility was weaker for 5a6a-EC and 5b6b-EC (r = 0.2 and r = 0.1, respectively)^42^, so the results for these oxysterols need to be interpreted in this context. We observed that the study region had a strong impact on concentrations of some non-enzymatically produced oxysterols, including 5a6a-EC, 5b6b-EC, and 7-KC, most likely due to differences in sample matrix (plasma vs. serum). Thus, we controlled for study region in all of the analyses. Despite careful sample handing and the use of validated protocols, there is some risk of measurement error, and we cannot rule out the possibility that oxysterol concentrations in blood samples may have been affected by long-term storage^43–45^. The CV of some oxysterols was relatively high, potentially leading to exposure misclassification. Given the number of factors investigated, it is possible that some of the results are due to multiple testing. This study is exploratory and associations need to be evaluated in future studies.

## Conclusion

The findings of this study suggest that cholesterol precursors are most strongly associated with metabolic factors and metabolic diseases including BMI and CVD, as well as with breast cancer tumor characteristics such as tumor stage and endocrine therapy. The oxysterols 7-KC, 7a-HC, and 7b-HC, formed via ROS, were associated with obesity, and THC and 7b-HC, were associated with breast cancer tumor characteristics. Weak associations with case-related characteristics were observed for the estrogen receptor modulating oxysterols 27-HC and 25-HC and the remaining oxysterols, and for lifestyle and reproductive characteristics with any of the oxysterols. Overall, however, the case-characteristics evaluated in this study accounted for a low proportion of the observed variance in oxysterol concentrations.

This cross-sectional study provides evidence for a number of patient and disease characteristics associated with circulating oxysterol concentrations in women with breast cancer. While this is the first study of its kind and the results need to be confirmed in other studies, a better characterization of circulating cholesterol precursors and oxysterols is of interest for past and future oxysterol studies in women with breast cancer. The results of this cross-sectional study suggest associations between cholesterol precursors and metabolic factors, while selected oxysterols were associated with breast cancer tumor characteristics, warranting further investigation of the role of cholesterol precursors and metabolic diseases, such as obesity and obesity-related diseases, and oxysterols and pathological processes in women with breast cancer and in other populations.

## Data availability

The datasets generated and analyzed during the current study are available from the corresponding author on reasonable request.

### Supplementary Information

Supplementary Information.

## References

[1] Mutemberezi V, Guillemot-Legris O, Muccioli GG (2016). Oxysterols: From cholesterol metabolites to key mediators. Prog. Lipid Res..

[2] Brown AJ, Sharpe LJ, Rogers MJ (2021). Oxysterols: From physiological tuners to pharmacological opportunities. Br. J. Pharmacol..

[3] Sottero B (2019). Lipid oxidation derived aldehydes and oxysterols between health and disease. Eur. J. Lipid Sci. Technol..

[4] Guillemot-Legris O, Mutemberezi V, Muccioli GG (2016). Oxysterols in metabolic syndrome: From bystander molecules to bioactive lipids. Trends Mol. Med..

[5] Kloudova A, Guengerich FP, Soucek P (2017). The role of oxysterols in human cancer. Trends Endocrinol. Metab. TEM.

[6] Griffiths WJ, Wang Y (2019). Oxysterol research: A brief review. Biochem. Soc. Trans..

[7] Lu DL (2019). Circulating 27-hydroxycholesterol and breast cancer risk: Results from the EPIC-Heidelberg cohort. J. Natl. Cancer Inst..

[8] Decker NS (2023). Endogenous Estrogen receptor modulating oxysterols and breast cancer prognosis: Results from the MARIE patient cohort. Br. J. Cancer.

[9] Kloudova-Spalenkova A (2020). Plasma oxysterol levels in luminal subtype breast cancer patients are associated with clinical data. J. Steroid Biochem. Mol. Biol..

[10] Decker NS (2023). Circulating oxysterols and prognosis among women with a breast cancer diagnosis: results from the MARIE patient cohort. BMC Med..

[11] Inasu M (2021). High CYP27A1 expression is a biomarker of favorable prognosis in premenopausal patients with estrogen receptor positive primary breast cancer. npj Breast Cancer.

[12] Kimbung S (2020). CYP27A1 expression is associated with risk of late lethal estrogen receptor-positive breast cancer in postmenopausal patients. Breast Cancer Res..

[13] Kimbung S (2017). Impact of 27-hydroxylase (CYP27A1) and 27-hydroxycholesterol in breast cancer. Endocrine-Relat. Cancer.

[14] Le Cornet C, Johnson TS, Lu DL, Kaaks R, Fortner RT (2020). Association between lifestyle, dietary, reproductive, and anthropometric factors and circulating 27-hydroxycholesterol in EPIC-Heidelberg. Cancer Causes Control.

[15] Brown AJ, Ikonen E, Olkkonen VM (2014). Cholesterol precursors: more than mere markers of biosynthesis. Curr. Opin. Lipidol..

[16] Zmyslowski A, Szterk A (2019). Oxysterols as a biomarker in diseases. Clin. Chim Acta.

[17] Luu W, Sharpe LJ, Capell-Hattam I, Gelissen IC, Brown AJ (2016). Oxysterols: Old tale, new twists. Ann. Rev. Pharmacol. Toxicol..

[18] Yamauchi Y, Rogers MA (2018). Sterol metabolism and transport in atherosclerosis and cancer. Front. Endocrinol..

[19] Gómez-Coronado D, Lasunción MA, Martínez-Botas J, Fernández-Suárez ME (2020). Role of cholesterol metabolism in the anticancer pharmacology of selective estrogen receptor modulators. Sem. Cancer Biol..

[20] Möhl A (2021). Comorbidity burden in long-term breast cancer survivors compared with a cohort of population-based controls from the MARIE study. Cancer.

[21] Buck K, Vrieling A, Flesch-Janys D, Chang-Claude J (2011). Dietary patterns and the risk of postmenopausal breast cancer in a German case-control study. Cancer Causes Control.

[22] Abbas S (2008). Serum 25-hydroxyvitamin D and risk of post-menopausal breast cancer–results of a large case-control study. Carcinogenesis.

[23] Vrieling A (2011). Serum 25-hydroxyvitamin D and postmenopausal breast cancer survival: a prospective patient cohort study. Breast Cancer Res..

[24] Rosner B (1983). Percentage points for a generalized ESD many-outlier procedure. Technometrics.

[25] Hansel B (2004). Metabolic syndrome is associated with elevated oxidative stress and dysfunctional dense high-density lipoprotein particles displaying impaired antioxidative activity. J. Clin. Endocrinol. Metab..

[26] Keaney JF (2003). Obesity and systemic oxidative stress: clinical correlates of oxidative stress in the Framingham Study. Arteriosclerosis Thrombosis Vasc. Biol..

[27] Lizard G (1998). Glutathione is implied in the control of 7-ketocholesterol-induced apoptosis, which is associated with radical oxygen species production. FASEB J..

[28] Lemaire-Ewing S (2005). Comparison of the cytotoxic, pro-oxidant and pro-inflammatory characteristics of different oxysterols. Cell Biol. Toxicol..

[29] Baila-Rueda L (2018). Cholesterol oversynthesis markers define familial combined hyperlipidemia versus other genetic hypercholesterolemias independently of body weight. J. Nutrit. Biochem..

[30] Baila-Rueda L (2014). Bile acid synthesis precursors in familial combined hyperlipidemia: the oxysterols 24S-hydroxycholesterol and 27-hydroxycholesterol. Biochem. Biophys. Res. Commun..

[31] Stiles AR (2014). Genetic, anatomic, and clinical determinants of human serum sterol and vitamin D levels. Proc. Natl. Acad. Sci. USA.

[32] Sato R (2010). Sterol metabolism and SREBP activation. Arch. Biochem. Biophys..

[33] Leignadier J, Dalenc F, Poirot M, Silvente-Poirot S (2017). Improving the efficacy of hormone therapy in breast cancer: The role of cholesterol metabolism in SERM-mediated autophagy, cell differentiation and death. Biochem. Pharmacol..

[34] Rossouw JE (2012). Relationships of coronary heart disease with 27-hydroxycholesterol, low-density lipoprotein cholesterol, and menopausal hormone therapy. Circulation.

[35] Dalenc F (2017). Circulating oxysterol metabolites as potential new surrogate markers in patients with hormone receptor-positive breast cancer: Results of the OXYTAM study. J. Steroid Biochem. Mol. Biol..

[36] Wu Q (2013). 27-Hydroxycholesterol promotes cell-autonomous ER-positive breast cancer growth. Cell Rep..

[37] Le Cornet, C. *et al.* Circulating 27-hydroxycholesterol and breast cancer tissue expression of CYP27A1 CYP7B1 LXR-β and ERβ: Results from the EPIC-Heidelberg cohort. *Breast Cancer Res.* **22**(1), 23. 10.1186/s13058-020-1253-6 (2020). 10.1186/s13058-020-1253-6 PMC7031866 32075687

[38] Tremblay-Franco M (2015). Effect of obesity and metabolic syndrome on plasma oxysterols and fatty acids in human. Steroids.

[39] Ornelas A (2017). Beyond COX-1: The effects of aspirin on platelet biology and potential mechanisms of chemoprevention. Cancer Metastasis Rev..

[40] Chen WY, Holmes MD (2017). Role of aspirin in breast cancer survival. Curr. Oncol. Rep..

[41] Passarelli MN (2022). Association of demographic and health characteristics with circulating oxysterol concentrations. J. Clin. Lipidol..

[42] Lu DL (2018). Reproducibility of serum oxysterols and lanosterol among postmenopausal women: Results from EPIC-Heidelberg. Clin. Biochem..

[43] Haid M (2018). Long-term stability of human plasma metabolites during storage at -80 °C. J. Proteome Res..

[44] Wagner-Golbs A, Neuber S, Kamlage B, Christiansen N, Bethan B, Rennefahrt U, Lind L (2019). Effects of long-term storage at− 80 C on the human plasma metabolome. Metabolites.

[45] Dzeletovic S, Breuer O, Lund E, Diczfalusy U (1995). Determination of cholesterol oxidation products in human plasma by isotope dilution-mass spectrometry. Anal. Biochem..

