# Supplementary material for: Associations between lifestyle, health, and clinical characteristics and circulating oxysterols and cholesterol precursors in women diagnosed with breast cancer: a cross-sectional study
Source: Sci Rep. 2024 Feb 29;14:4977. doi: 10.1038/s41598-024-55316-x (PMC10904394; doi:10.1038/s41598-024-55316-x)
Supplement: Supplementary file 1 — Supplementary Information. [file 41598_2024_55316_MOESM1_ESM.docx]

# Supplemental Material

## Oxysterol metabolism: Background

Oxysterols including 27-HC, 25-HC, 5a,6a-EC, 5b,6b-EC, 7a-HC, 7b-HC, 7-KC, THC are oxidized derivates of cholesterol, whereas the sterols lanosterol, 24-DHLan, desmosterol, and 7-DC, are precursors of cholesterol ^1,2^.

In the upstream cholesterol pathway, lanosterol represents an early metabolite in the endogenous cholesterol synthesis. After lanosterol, the cholesterol synthesis is split into Bloch pathway including desmosterol and Kandutsch-Russel pathway including 24-DHLan and 7-DC. In addition to its role as cholesterol precursor, 7-DC also represents an intermediate in the vitamin D synthesis ^1,3^.

The oxysterols 27-HC, 25-HC, 5a,6a-EC, 5b,6b-EC, 7a-HC, and 7b-HC are primary downstream metabolites of cholesterol, while 7-KC and THC represent secondary metabolites ^4^. Oxysterols can either be produced enzymatically including 27-HC and 24S-HC, which are converted by enzymes of the cytochrome P450 family ^5,6^, or non-enzymatically, including the oxysterols 7b-HC, 5a6a-EC, 5b6b-EC, and THC, which are produced via reactive oxysterol species (ROS) ^1^. Some oxysterols including 25-HC, 7a-HC, and 7-KC can be metabolized enzymatically and non-enzymatically ^1^.

### Table S1: Table of participants’ characteristics (continuous)

| **Baseline Characteristics** | **n (total)** | **Median (range) or n (%)** |
| --- | --- | --- |
| Age at diagnosis in years | 2282 | 63.0 (50.0-75.0) |
| BMI (continuous) in kg/m^2^ | 2279 | 25.3 (15.5-49.6) |
| Time between diagnosis and blood collection in months* | 2282 | 3.7 (-14.5– 57.6) |

^*^eight participants with blood collection before breast cancer diagnosis (due to original recruitment as “control”, then re-classification as “case”). Previously published in: ^7^

### Table S2: Analyte concentrations of circulating oxysterols and coefficients of variation (CV%)

| Analyte (nM) | n | Median (range) study population in nM | Inter-assay CV % | Intra-assay CV % |
| --- | --- | --- | --- | --- |
| *Exposure analytes* | |  |  |  |
| 24S-HC | 2280 | 94.3 (17.2-1973) | 20.2 % | 7.9 % |
| 7a-HC | 2271 | 272.2 (25.6-5458) | 17.3% | 9.5 % |
| 7b-HC | 2272 | 201.2 (1.3-6061) | 74.9% | < LOD |
| 7-KC | 2282 | 184.9 (10.1-106000) | 42.4% | 9.1 % |
| 5a6a-EC | 2281 | 26.3 (1.8-4559) | 31.8 % | 18.4 % |
| 5b6b-EC | 2278 | 101.3 (5.9-10228) | 20.0% | 7.6 % |
| THC | 2282 | 7.1 (0.3-758) | 41.3% | < LOD |
| 7-DC | 2221 | 541.7 (9.6-10252) | 18.3% | 9.8 % |
| 24-DHLan* | 1027 | 40.1 (5.6-413) | 33.7 % | < LOD |
| Lan | 2282 | 545.2 (57.8-3256) | 20.7% | 14.2 % |
| Desmos | 2281 | 1875.0 (51.9-10643) | 20.2% | 6.5 % |
| 22R-HC* | 281 | 6.4 (1.4-142) | < LOD | < LOD |
| 24, 25-EC* | 90 | 11.5 (5.8-105) | < LOD | < LOD |
| *Covariate analytes* | |  |  |  |
| 27-HC | 2282 | 210.0 (85.6-600) | 15.8% | 7.5 % |
| 25-HC | 2282 | 20.4 (2.5-5719) | 29.2% | 16.0 % |
| Estradiol** | 2282 | 0.08 (0.0-4.3) | 16.2% | 13.8% |

N= total number of biomarker values including imputed values and excluding values >calibration range; unit: nM=nanomolar
values below the limit of detection (LOD) imputed with the midpoint between 0 and the lowest detectable value.
values exceeding calibration range, excluded from main analysis

*No imputation due to high proportion (>50%) of values below LOD
**Estradiol: 0.08 (0.0-4.3) nM corresponding to 22.1 (0.0-1175) pg/ml

Abbreviations: 24S-HC=24S-hydroxycholesterol; 5a6a-EC=5α,6α-epoxycholesterol; 5b6b-EC=5β,6β-epoxycholesterol; 7-KC=7-ketocholesterol; 7a-HC=7α-hydroxycholesterol; 7b-HC=7β-hydroxycholesterol; Lan=lanosterol; 24-DHLan=24,25-dihydrolanosterol; 7-DC=7-dehydrocholesterol; Desmos=desmosterol; THC=5α,6β-dihydroxycholestanol; 24,25-EC=24,25-epoxycholesterol; 22R-HC=22R- hydroxycholesterol; 27-HC=27-hydroxycholesterol; 25-HC=25-hydroxycholesterol.

Previously published in: ^8^.

### Table S3: Additional cross-sectional associations between anthropometric, lifestyle, and reproductive characteristics and oxysterol concentrations by metabolic pathway

| **Exposure** | | **Categories** | | **n (%)^a^** | | **Geometric mean  (95% CI), nM** | | **% diff** | | **Geometric mean  (95% CI), nM** | | **% diff** | | |  | **Geometric mean  (95% CI), nM** | **% diff** | | **Geometric mean  (95% CI), nM** | | | **% diff** |
| --- | --- | --- | --- | --- | --- | --- | --- | --- | --- | --- | --- | --- | --- | --- | --- | --- | --- | --- | --- | --- | --- | --- |
| **Cholesterol precursors** | | | |  | | **Lanosterol  (n=2282)** | |  | | **24-DHLan  (n=1027)** | |  | |  | **Desmosterol (n=2281)** | |  | **7-DC (n=2221)** | | |  | |
| Smoking | | No | | 1239 (54.3) | | 546.1 (531.5,561.1) | | Ref. | | 39.7 (37.8,41.6) | | Ref. | | | 1732.4 (1683.3,1782.9) | | Ref. | | 508.6 (477.4,541.9) | Ref. | | |
|  | | Former | | 615 (27.0) | | 508.3 (489.5,527.8) | | -6.93 | | 39.3 (36.8,41.9) | | -1.01 | | | 1668.8 (1603.7,1736.7) | | -3.67 | | 491.7 (450.3,536.9) | -3.34 | | |
|  | | Current | | 428 (18.8) | | 538.0 (513.6,563.4) | | -1.49 | | 40.6 (37.6,43.8) | | 2.22 | | | 1688.4 (1607.7,1773.2) | | -2.54 | | 468.1 (420.6,520.9) | -7.98 | | |
| p-value, extreme categories | |  | |  | |  | | 0.589 | |  | | 0.643 | | |  | | 0.383 | |  | 0.120 | | |
| Alcohol consumption (g/day) | | None | | 540 (23.7) | | 522.6 (501.8,544.2) | | Ref. | | 40.0 (37.2,42.9) | | Ref. | | | 1692.1 (1621.2,1766.1) | | Ref. | | 493.3 (449.0,542.0) | Ref. | | |
|  | | <19 | | 1405 (61.6) | | 540.0 (526.7,553.7) | | 3.34 | | 39.1 (37.4,40.8) | | -2.29 | | | 1714.5 (1669.8,1760.4) | | 1.33 | | 495.3 (467.4,524.9) | 0.40 | | |
|  | | 19+ | | 335 (14.7) | | 528.9 (502.3,556.9) | | 1.21 | | 42.2 (38.8,46.0) | | 5.57 | | | 1697.2 (1607.2,1792.2) | | 0.30 | | 509.3 (452.0,573.7) | 3.23 | | |
| p-value, extreme categories | |  | |  | |  | | 0.720 | |  | | 0.338 | | |  | | 0.932 | |  | 0.683 | | |
| Leisure time physical activity (met*h/week) | | < 28 | | 653 (28.6) | | 528.7 (509.2,548.9) | | Ref. | | 40.7 (38.2,43.3) | | Ref. | | | 1588.7 (1527.1,1652.7) | | Ref. | | 494.7 (453.2,539.9) | Ref. | | |
|  | | ≥28 | | 1629 (71.4) | | 536.1 (523.7,548.6) | | 1.39 | | 39.4 (37.8,41.0) | | -3.19 | | | 1754.3 (1712.0,1797.7) | | 10.43 | | 496.5 (470.4,524.0) | 0.37 | | |
|  | |  | |  | |  | | 0.542 | |  | | 0.394 | | |  | | <.0001 | |  | 0.944 | | |
| Menopausal hormone therapy | | No | | 1231 (53.9) | | 539.7 (525.3,554.5) | | Ref. | | 40.9 (39.1,42.8) | | Ref. | | | 1709.4 (1661.2,1759.0) | | Ref. | | 495.9 (465.5,528.1) | Ref. | | |
|  | | Yes | | 1038 (45.5) | | 527.8 (512.5,543.5) | | -2.21 | | 38.4 (36.5,40.4) | | -6.09 | | | 1705.3 (1653.3,1759.0) | | -0.24 | | 497.1 (464.4,532.1) | 0.25 | | |
| p-value, extreme categories | |  | |  | |  | | 0.278 | |  | | 0.076 | | |  | | 0.914 | |  | 0.958 | | |
| Estradiol level (nM) | | <0.08 | | 1139 (49.9) | | 560.9 (545.5,576.8) | | Ref. | | 40.2 (38.3,42.1) | | Ref. | | | 1747.4 (1696.4,1799.8) | | Ref. | | 502.3 (470.6,536.1) | Ref. | | |
|  | | ≥0.08 | | 1143 (50.1) | | 508.8 (494.9,523.1) | | -9.29 | | 39.3 (37.4,41.2) | | -2.27 | | | 1667.2 (1619.1,1716.8) | | -4.59 | | 489.9 (459.3,522.5) | -2.47 | | |
| p-value, extreme categories | |  | |  | |  | | <.0001 | |  | | 0.508 | | |  | | 0.028 | |  | 0.595 | | |
| Vitamin D level (nmol/L) | | 9.7-35.5 | | 367 (16.1) | | 565.6 (537.9,594.8) | | Ref. | | 40.5 (37.1,44.1) | | Ref. | | | 1762.1 (1672.9,1856.0) | | Ref. | | 614.7 (561.2,673.3) | Ref. | | |
|  | | 35.6-57.0 | | 369 (16.2) | | 555.8 (529.1,583.8) | | -1.74 | | 41.2 (37.9,44.9) | | 1.82 | | | 1668.2 (1585.6,1755.1) | | -5.33 | | 616.6 (564.1,674.1) | 0.32 | | |
|  | | 57.1-240.3 | | 367 (16.1) | | 546.6 (519.8,574.7) | | -3.37 | | 43.1 (39.3,47.2) | | 6.37 | | | 1761.6 (1672.7,1855.3) | | -0.03 | | 559.1 (510.6,612.2) | -9.04 | | |
| p-value, extreme categories | |  | |  | |  | | 0.352 | |  | | 0.346 | | |  | | 0.995 | |  | 0.155 | | |
| Cholesterol intake (g/day) | | <208 | | 670 (29.4) | | 519.5 (501.2,538.5) | | Ref. | | 39.4 (37.0,42.0) | | Ref. | | | 1696.4 (1632.8,1762.4) | | Ref. | | 485.8 (447.4,527.5) | Ref. | | |
|  | | 208-283 | | 672 (29.4) | | 539.9 (520.9,559.5) | | 3.92 | | 37.9 (35.6,40.2) | | -4.04 | | | 1687.7 (1624.8,1753.1) | | -0.51 | | 512.7 (472.4,556.5) | 5.55 | | |
|  | | ≥283 | | 672 (29.4) | | 542.0 (523.0,561.8) | | 4.34 | | 41.5 (38.9,44.2) | | 5.19 | | | 1764.8 (1698.8,1833.4) | | 4.04 | | 495.2 (456.0,537.7) | 1.93 | | |
| p-value, extreme categories | |  | |  | |  | | 0.101 | |  | | 0.270 | | |  | | 0.151 | |  | 0.747 | | |
| Energy intake (kcal/day) | | <1507 | | 670 (29.4) | | 523.6 (505.2,542.7) | | Ref. | | 39.8 (37.4,42.4) | | Ref. | | | 1661.9 (1599.9,1726.4) | | Ref. | | 505.4 (465.6,548.6) | Ref. | | |
|  | | 1507-1905 | | 672 (29.4) | | 538.0 (519.1,557.5) | | 2.74 | | 38.3 (36.0,40.7) | | -3.77 | | | 1722.5 (1658.2,1789.2) | | 3.64 | | 481.7 (443.6,523.0) | -4.7 | | |
|  | | ≥1905 | | 672 (29.4) | | 539.8 (520.8,559.4) | | 3.09 | | 40.5 (38.0,43.1) | | 1.73 | | | 1764.9 (1699.1,1833.2) | | 6.19 | | 506.6 (466.8,549.8) | 0.23 | | |
| p-value, extreme categories | |  | |  | |  | | 0.237 | |  | | 0.705 | | |  | | 0.028 | |  | 0.747 | | |
| Waist-Hip-Ratio^c^ | | <0.8 | | 710 (31.1) | | 524.3 (505.8,543.6) | | Ref. | | 39.6 (37.2,42.0) | | Ref. | | | 1728.9 (1664.4,1795.9) | | Ref. | | 482.6 (444.1,524.4) | Ref. | | |
|  | | 0.80-0.84 | | 711 (31.2) | | 527.5 (509.3,546.4) | | 0.6 | | 38.3 (36.0,40.7) | | -3.32 | | | 1705.8 (1643.7,1770.3) | | -1.34 | | 515.4 (475.0,559.1) | 6.8 | | |
|  | | ≥0.85 | | 861 (37.7) | | 547.8 (530.1,566.1) | | 4.47 | | 41.2 (38.9,43.6) | | 4.11 | | | 1688.3 (1630.6,1748.0) | | -2.35 | | 491.6 (455.2,530.9) | 1.87 | | |
| p-value, extreme categories | |  | |  | |  | | 0.086 | |  | | 0.357 | | |  | | 0.377 | |  | 0.755 | | |
| Study region | | Hamburg | | 1047 (45.9) | | 514.5 (499.8,529.6) | | Ref. | | 38.0 (36.2,39.9) | | Ref. | | | 1675.6 (1625.1,1727.7) | | Ref. | | 421.7 (394.2,451.0) | Ref. | | |
|  | | Rhine-Neckar-Karlsruhe | | 1235 (54.1) | | 554.3 (539.7,569.3) | | 7.74 | | 41.6 (39.7,43.6) | | 9.50 | | | 1737.9 (1689.6,1787.6) | | 3.72 | | 583.4 (548.3,620.8) | 38.36 | | |
| p-value, extreme categories | |  | |  | |  | | 0.0002 | |  | | 0.008 | | |  | | 0.086 | |  | <.0001 | | |
| **Cholesterol metabolites: enzymatic pathway^b^** | | | |  | | **27-HC (n=2282)** | |  | | **25-HC (n=2282)** | |  | | | **24S-HC (n=2279)** | |  | |  |  |  |  |
| Smoking | | No | | 1239 (54.3) | | 209.7 (206.8,212.7) | | Ref. | | 20.2 (19.5,20.9) | | Ref. | | | 97.1 (95.3,98.9) | | Ref. | |  |  |  |  |
|  | | Former | | 615 (27.0) | | 207.4 (203.4,211.4) | | -1.11 | | 19.1 (18.2,20.0) | | -5.41 | | | 91.8 (89.5,94.2) | | -5.42 | |  |  |  |  |
|  | | Current | | 428 (18.8) | | 211.1 (206.2,216.2) | | 0.69 | | 20.1 (19.0,21.3) | | -0.43 | | | 90.8 (88.0,93.7) | | -6.47 | |  |  |  |  |
| p-value, extreme categories | |  | |  | |  | | 0.633 | |  | | 0.903 | | |  | | 0.0005 | |  |  |  |  |
| Alcohol consumption (g/day) | | None | | 540 (23.7) | | 203.4 (199.2,207.7) | | Ref. | | 19.5 (18.6,20.6) | | Ref. | | | 93.3 (90.7,95.9) | | Ref. | |  |  |  |  |
|  | | <19 | | 1405 (61.6) | | 210.6 (207.9,213.3) | | 3.53 | | 20.0 (19.4,20.7) | | 2.54 | | | 95.6 (94.0,97.3) | | 2.47 | |  |  |  |  |
|  | | 19+ | | 335 (14.7) | | 214.1 (208.5,219.9) | | 5.28 | | 19.8 (18.5,21.1) | | 1.29 | | | 91.4 (88.2,94.7) | | -1.97 | |  |  |  |  |
| p-value, extreme categories | |  | |  | |  | | 0.003 | |  | | 0.763 | | |  | | 0.389 | |  |  |  |  |
| Leisure time physical activity (met*h/week) | | < 28 | | 653 (28.6) | | 207.0 (203.0,211.0) | | Ref. | | 19.5 (18.6,20.4) | | Ref. | | | 92.7 (90.4,95.2) | | Ref. | |  |  |  |  |
|  | | ≥28 | | 1629 (71.4) | | 210.3 (207.8,212.8) | | 1.59 | | 20.0 (19.4,20.6) | | 2.77 | | | 95.1 (93.6,96.6) | | 2.53 | |  |  |  |  |
| p-value, extreme categories | |  | |  | |  | | 0.176 | |  | | 0.340 | | |  | | 0.110 | |  |  |  |  |
| Menopausal hormone therapy | | No | | 1231 (53.9) | | 210.3 (207.4,213.3) | | Ref. | | 20.1 (19.4,20.8) | | Ref. | | | 95.0 (93.2,96.8) | | Ref. | |  |  |  |  |
|  | | Yes | | 1038 (45.5) | | 208.1 (204.9,211.2) | | -1.08 | | 19.6 (18.9,20.4) | | -2.04 | | | 93.7 (91.8,95.6) | | -1.36 | |  |  |  |  |
| p-value, extreme categories | |  | |  | |  | | 0.308 | |  | | 0.430 | | |  | | 0.334 | |  |  |  |  |
| Estradiol level (nM) | | <0.08 | | 1139 (49.9) | | 212.8 (209.8,215.9) | | Ref. | | 19.7 (19.1,20.5) | | Ref. | | | 95.9 (94.1,97.8) | | Ref. | |  |  |  |  |
|  | | ≥0.08 | | 1143 (50.1) | | 206.0 (203.0,208.9) | | -3.24 | | 20.0 (19.3,20.7) | | 1.35 | | | 93.0 (91.2,94.8) | | -3.08 | |  |  |  |  |
| p-value, extreme categories | |  | |  | |  | | 0.002 | |  | | 0.602 | | |  | | 0.025 | |  |  |  |  |
| Vitamin D level (nmol/L) | | 9.7-35.5 | | 367 (16.1) | | 212.1 (207.1,217.3) | | Ref. | | 17.0 (15.9,18.1) | | Ref. | | | 96.5 (93.4,99.7) | | Ref. | |  |  |  |  |
|  | | 35.6-57.0 | | 369 (16.2) | | 214.5 (209.6,219.6) | | 1.13 | | 16.6 (15.6,17.6) | | -2.27 | | | 96.9 (93.9,100.1) | | 0.48 | |  |  |  |  |
|  | | 57.1-240.3 | | 367 (16.1) | | 209.9 (205.0,215.0) | | -1.04 | | 16.4 (15.4,17.4) | | -3.35 | | | 95.6 (92.6,98.8) | | -0.85 | |  |  |  |  |
| p-value, extreme categories | |  | |  | |  | | 0.552 | |  | | 0.457 | | |  | | 0.721 | |  |  |  |  |
| Cholesterol intake (g/day) | | <208 | | 670 (29.4) | | 209.3 (205.5,213.3) | | Ref. | | 19.9 (19.0,20.9) | | Ref. | | | 96.1 (93.7,98.5) | | Ref. | |  |  |  |  |
|  | | 208-283 | | 672 (29.4) | | 210.2 (206.3,214.1) | | 0.41 | | 20.4 (19.5,21.4) | | 2.39 | | | 95.1 (92.8,97.5) | | -1.02 | |  |  |  |  |
|  | | ≥283 | | 672 (29.4) | | 209.0 (205.2,212.9) | | -0.16 | | 19.1 (18.3,20.0) | | -4.12 | | | 92.5 (90.2,94.8) | | -3.78 | |  |  |  |  |
| p-value, extreme categories | |  | |  | |  | | 0.108 | |  | | 0.207 | | |  | | 0.032 | |  |  |  |  |
| Energy intake (kcal/day) | | <1507 | | 670 (29.4) | | 206.8 (203.0,210.6) | | Ref. | | 19.9 (19.0,20.8) | | Ref. | | | 96.0 (93.6,98.4) | | Ref. | |  |  |  |  |
|  | | 1507-1905 | | 672 (29.4) | | 210.6 (206.7,214.5) | | 1.83 | | 19.8 (18.9,20.7) | | -0.21 | | | 94.6 (92.3,97.0) | | -1.45 | |  |  |  |  |
|  | | ≥1905 | | 672 (29.4) | | 211.2 (207.4,215.2) | | 2.16 | | 19.8 (18.9,20.7) | | -0.25 | | | 93.0 (90.8,95.4) | | -3.08 | |  |  |  |  |
| p-value, extreme categories | |  | |  | |  | | 0.906 | |  | | 0.941 | | |  | | 0.081 | |  |  |  |  |
| Waist-Hip-Ratio^c^ | | <0.80 | | 710 (31.1) | | 209.5 (205.7,213.5) | | Ref. | | 19.8 (18.9,20.7) | | Ref. | | | 93.1 (90.8,95.4) | | Ref. | |  |  |  |  |
|  | | 0.80-0.84 | | 711 (31.2) | | 208.0 (204.3,211.8) | | -0.71 | | 19.4 (18.6,20.3) | | -1.85 | | | 94.8 (92.5,97.1) | | 1.88 | |  |  |  |  |
|  | | ≥0.85 | | 861 (37.7) | | 210.3 (206.7,213.8) | | 0.34 | | 20.3 (19.5,21.2) | | 2.54 | | | 95.2 (93.1,97.4) | | 2.31 | |  |  |  |  |
| p-value, extreme categories | |  | |  | |  | | 0.793 | |  | | 0.437 | | |  | | 0.195 | |  |  |  |  |
| Study region | | Hamburg | | 1047 (45.9) | | 207.8 (204.7,210.9) | | Ref. | | 23.7 (22.8,24.5) | | Ref. | | | 93.1 (91.3,95.0) | | Ref. | |  |  |  |  |
|  | | Rhine-Neckar-Karlsruhe | | 1235 (54.1) | | 210.9 (208.0,213.8) | | 1.48 | | 16.7 (16.1,17.3) | | -29.40 | | | 95.7 (94.0,97.5) | | 2.83 | |  |  |  |  |
| p-value, extreme categories | |  | |  | |  | | 0.157 | |  | | <.0001 | | |  | | 0.045 | |  |  |  |  |
| **Cholesterol metabolites: non- enzymatic pathway^b^** | | | |  | | **5a6a-EC  (n=2281)** | |  | | **5b6b-EC  (n=2278)** | |  | | | **THC (n=2282)** | |  | |  |  |  |  |
| Smoking | | No | | 1239 (54.3) | | 28.1 (27.2,29.1) | | Ref. | | 110.4 (106.9,114.1) | | Ref. | | | 4.8 (4.5,5.2) | | Ref. | |  |  |  |  |
|  | | Former | | 615 (27.0) | | 27.2 (26.0,28.5) | | -3.21 | | 108.8 (104.0,113.8) | | -1.51 | | | 4.2 (3.8,4.7) | | -12.40 | |  |  |  |  |
|  | | Current | | 428 (18.8) | | 28.6 (27.1,30.3) | | 1.77 | | 113.1 (107.0,119.5) | | 2.40 | | | 4.8 (4.2,5.4) | | -0.31 | |  |  |  |  |
| p-value, extreme categories | |  | |  | |  | | 0.601 | |  | | 0.477 | | |  | | 0.967 | |  |  |  |  |
| Alcohol consumption (g/day) | | None | | 540 (23.7) | | 27.3 (26.0,28.7) | | Ref. | | 108.9 (103.8,114.3) | | Ref. | | | 4.6 (4.1,5.2) | | Ref. | |  |  |  |  |
|  | | <19 | | 1405 (61.6) | | 28.2 (27.4,29.1) | | 3.37 | | 110.9 (107.7,114.3) | | 1.84 | | | 4.7 (4.4,5.1) | | 2.26 | |  |  |  |  |
|  | | 19+ | | 335 (14.7) | | 28.1 (26.4,29.9) | | 2.98 | | 111.5 (104.8,118.6) | | 2.34 | | | 4.2 (3.7,4.8) | | -9.05 | |  |  |  |  |
| p-value, extreme categories | |  | |  | |  | | 0.470 | |  | | 0.565 | | |  | | 0.298 | |  |  |  |  |
| Leisure time physical activity (met*h/week) | | < 28 | | 653 (28.6) | | 27.9 (26.7,29.2) | | Ref. | | 109.4 (104.6,114.5) | | Ref. | | | 4.8 (4.3,5.3) | | Ref. | |  |  |  |  |
|  | | ≥28 | | 1629 (71.4) | | 28.0 (27.2,28.8) | | 0.33 | | 110.9 (107.8,114.0) | | 1.32 | | | 4.6 (4.3,4.9) | | -4.11 | |  |  |  |  |
| p-value, extreme categories | |  | |  | |  | | 0.904 | |  | | 0.628 | | |  | | 0.494 | |  |  |  |  |
| Menopausal hormone therapy | | No | | 1231 (53.9) | | 27.7 (26.8,28.6) | | Ref. | | 109.9 (106.4,113.6) | | Ref. | | | 4.8 (4.5,5.2) | | Ref. | |  |  |  |  |
|  | | Yes | | 1038 (45.5) | | 28.3 (27.3,29.3) | | 2.37 | | 111.1 (107.3,115.1) | | 1.08 | | | 4.4 (4.0,4.7) | | -9.47 | |  |  |  |  |
| p-value, extreme categories | |  | |  | |  | | 0.346 | |  | | 0.661 | | |  | | 0.075 | |  |  |  |  |
| Estradiol level (nM) | | <0.08 | | 1139 (49.9) | | 28.0 (27.1,29.0) | | Ref. | | 109.2 (105.6,113.0) | | Ref. | | | 4.6 (4.2,4.9) | | Ref. | |  |  |  |  |
|  | | ≥0.08 | | 1143 (50.1) | | 27.9 (27.0,28.9) | | -0.32 | | 111.7 (108.1,115.5) | | 2.27 | | | 4.7 (4.3,5.1) | | 2.44 | |  |  |  |  |
| p-value, extreme categories | |  | |  | |  | | 0.895 | |  | | 0.353 | | |  | | 0.660 | |  |  |  |  |
| Vitamin D level (nmol/L) | | 9.7-35.5 | | 367 (16.1) | | 18.6 (17.6,19.7) | | Ref. | | 68.3 (64.5,72.3) | | Ref. | | | 3.0 (2.6,3.5) | | Ref. | |  |  |  |  |
|  | | 35.6-57.0 | | 369 (16.2) | | 18.6 (17.6,19.6) | | -0.43 | | 67.9 (64.2,71.8) | | -0.57 | | | 2.6 (2.2,3.0) | | -15.23 | |  |  |  |  |
|  | | 57.1-240.3 | | 367 (16.1) | | 17.1 (16.2,18.1) | | -8.24 | | 62.0 (58.6,65.7) | | -9.19 | | | 2.7 (2.3,3.1) | | -11.58 | |  |  |  |  |
| p-value, extreme categories | |  | |  | |  | | 0.033 | |  | | 0.022 | | |  | | 0.299 | |  |  |  |  |
| Cholesterol intake (g/day) | | <208 | | 670 (29.4) | | 28.4 (27.2,29.7) | | Ref. | | 111.5 (106.8,116.5) | | Ref. | | | 4.5 (4.1,5.0) | | Ref. | |  |  |  |  |
|  | | 208-283 | | 672 (29.4) | | 28.6 (27.3,29.9) | | 0.59 | | 113.8 (109.0,118.9) | | 2.05 | | | 4.7 (4.3,5.2) | | 3.89 | |  |  |  |  |
|  | | ≥283 | | 672 (29.4) | | 27.5 (26.3,28.8) | | -3.16 | | 108.3 (103.7,113.2) | | -2.86 | | | 4.6 (4.1,5.0) | | 0.35 | |  |  |  |  |
| p-value, extreme categories | |  | |  | |  | | 0.317 | |  | | 0.357 | | |  | | 0.962 | |  |  |  |  |
| Energy intake (kcal/day) | | <1507 | | 670 (29.4) | | 28.2 (26.9,29.4) | | Ref. | | 111.1 (106.4,116.1) | | Ref. | | | 4.5 (4.1,5.0) | | Ref. | |  |  |  |  |
|  | | 1507-1905 | | 672 (29.4) | | 27.8 (26.6,29.1) | | -1.28 | | 109.2 (104.6,114.1) | | -1.72 | | | 4.6 (4.1,5.0) | | 1.13 | |  |  |  |  |
|  | | ≥1905 | | 672 (29.4) | | 28.5 (27.3,29.8) | | 1.34 | | 113.4 (108.5,118.4) | | 2.02 | | | 4.8 (4.3,5.3) | | 6.07 | |  |  |  |  |
| p-value, extreme categories | |  | |  | |  | | 0.676 | |  | | 0.524 | | |  | | 0.408 | |  |  |  |  |
| Waist-Hip-Ratio^c^ | <0.80 | | 710 (31.1) | | 27.4 (26.2,28.6) | | Ref. | | 108.3 (103.8,113.1) | | Ref. | | 4.5 (4.1,5.0) | | | | Ref. | |  |  |  |  |
|  | 0.80-0.84 | | 711 (31.2) | | 27.6 (26.4,28.8) | | 0.67 | | 108.9 (104.5,113.6) | | 0.58 | | 4.6 (4.2,5.1) | | | | 1.59 | |  |  |  |  |
|  | ≥0.85 | | 861 (37.7) | | 28.8 (27.6,29.9) | | 4.89 | | 113.6 (109.3,118.2) | | 4.92 | | 4.7 (4.3,5.2) | | | | 4.52 | |  |  |  |  |
| p-value, extreme categories |  | |  | |  | | 0.121 | |  | | 0.115 | |  | | | | 0.523 | |  |  |  |  |
| Study region | Hamburg | | 1047 (45.9) | | 43.6 (42.1,45.1) | | Ref. | | 187.3 (181.0,193.9) | | Ref. | | 8.2 (7.6,8.9) | | | | Ref. | |  |  |  |  |
|  | Rhine-Neckar-Karlsruhe | | 1235 (54.1) | | 17.9 (17.4,18.5) | | -58.85 | | 65.2 (63.1,67.3) | | -65.22 | | 2.6 (2.4,2.8) | | | | -68.36 | |  |  |  |  |
| p-value, extreme categories |  | |  | |  | | <.0001 | |  | | <.0001 | |  | | | | <.0001 | |  |  |  |  |
| **Cholesterol metabolites: non- enzymatic pathway^b^** | | | |  | | **7a-HC (n=2271)** | |  | | **7b-HC (n=2272)** | |  | | | **7-KC (n=2282)** | |  | |  |  |  |  |
| Smoking | | No | | 1239 (54.3) | | 290.5 (281.2,300.2) | | Ref. | | 187.2 (177.7,197.4) | | Ref. | | | 209.2 (199.5,219.5) | | Ref. | |  |  |  |  |
|  | | Former | | 615 (27.0) | | 282.5 (270.1,295.5) | | -2.77 | | 187.3 (174.2,201.4) | | 0.03 | | | 198.4 (185.7,212.0) | | -5.18 | |  |  |  |  |
|  | | Current | | 428 (18.8) | | 292.8 (277.0,309.5) | | 0.78 | | 188.6 (172.5,206.3) | | 0.73 | | | 208.5 (192.2,226.2) | | -0.34 | |  |  |  |  |
| p-value, extreme categories | |  | |  | |  | | 0.816 | |  | | 0.892 | | |  | | 0.944 | |  |  |  |  |
| Alcohol consumption (g/day) | | None | | 540 (23.7) | | 284.1 (270.8,298.2) | | Ref. | | 185.3 (171.4,200.3) | | Ref. | | | 201.4 (187.6,216.2) | | Ref. | |  |  |  |  |
|  | | <19 | | 1405 (61.6) | | 286.5 (278.1,295.2) | | 0.84 | | 185.2 (176.5,194.4) | | -0.03 | | | 207.6 (198.7,216.9) | | 3.11 | |  |  |  |  |
|  | | 19+ | | 335 (14.7) | | 307.3 (289.0,326.8) | | 8.15 | | 202.0 (182.9,223.1) | | 9.01 | | | 208.6 (190.6,228.4) | | 3.60 | |  |  |  |  |
| p-value, extreme categories | |  | |  | |  | | 0.050 | |  | | 0.183 | | |  | | 0.548 | |  |  |  |  |
| Leisure physical activity (met*h/week) | | < 28 | | 653 (28.6) | | 289.6 (276.9,302.8) | | Ref. | | 192.0 (178.6,206.4) | | Ref. | | | 208.7 (195.4,222.9) | | Ref. | |  |  |  |  |
|  | | ≥28 | | 1629 (71.4) | | 288.4 (280.6,296.6) | | -0.38 | | 185.8 (177.7,194.3) | | -3.22 | | | 205.1 (196.9,213.6) | | -1.74 | |  |  |  |  |
| p-value, extreme categories | |  | |  | |  | | 0.887 | |  | | 0.452 | | |  | | 0.658 | |  |  |  |  |
| Menopausal hormone therapy | | No | | 1231 (53.9) | | 276.6 (267.8,285.7) | | Ref. | | 180.6 (171.4,190.3) | | Ref. | | | 201.0 (191.7,210.8) | | Ref. | |  |  |  |  |
|  | | Yes | | 1038 (45.5) | | 304.1 (293.6,314.9) | | 9.95 | | 196.0 (185.2,207.4) | | 8.51 | | | 212.0 (201.3,223.2) | | 5.47 | |  |  |  |  |
| p-value, extreme categories | |  | |  | |  | | 0.0001 | |  | | 0.040 | | |  | | 0.141 | |  |  |  |  |
| Estradiol level (nM) | | <0.08 | | 1139 (49.9) | | 290.9 (281.3,300.8) | | Ref. | | 187.0 (177.1,197.4) | | Ref. | | | 202.4 (192.7,212.6) | | Ref. | |  |  |  |  |
|  | | ≥0.08 | | 1143 (50.1) | | 286.7 (277.4,296.4) | | -1.42 | | 188.0 (178.2,198.4) | | 0.56 | | | 209.8 (199.8,220.2) | | 3.62 | |  |  |  |  |
| p-value, extreme categories | |  | |  | |  | | 0.553 | |  | | 0.887 | | |  | | 0.315 | |  |  |  |  |
| Vitamin D level (nmol/L) | | 9.7-35.5 | | 367 (16.1) | | 212.6 (200.7,225.3) | | Ref. | | 91.5 (82.0,102.1) | | Ref. | | | 101.6 (93.4,110.4) | | Ref. | |  |  |  |  |
|  | | 35.6-57.0 | | 369 (16.2) | | 199.5 (188.5,211.2) | | -6.16 | | 92.5 (83.1,102.9) | | 1.06 | | | 103.2 (95.1,111.9) | | 1.59 | |  |  |  |  |
|  | | 57.1-240.3 | | 367 (16.1) | | 189.5 (178.9,200.8) | | -10.87 | | 90.3 (80.9,100.7) | | -1.33 | | | 91.6 (84.3,99.6) | | -9.80 | |  |  |  |  |
| p-value, extreme categories | |  | |  | |  | | 0.007 | |  | | 0.867 | | |  | | 0.092 | |  |  |  |  |
| Cholesterol intake (g/day) | | <208 | | 670 (29.4) | | 296.8 (284.2,310.0) | | Ref. | | 190.7 (177.6,204.7) | | Ref. | | | 213.0 (199.9,227.1) | | Ref. | |  |  |  |  |
|  | | 208-283 | | 672 (29.4) | | 291.0 (278.7,303.9) | | -1.94 | | 189.6 (176.6,203.4) | | -0.57 | | | 211.2 (198.2,225.1) | | -0.86 | |  |  |  |  |
|  | | ≥283 | | 672 (29.4) | | 282.8 (270.8,295.3) | | -4.72 | | 185.7 (173.0,199.3) | | -2.59 | | | 200.8 (188.3,214.0) | | -5.76 | |  |  |  |  |
| p-value, extreme categories | |  | |  | |  | | 0.123 | |  | | 0.608 | | |  | | 0.198 | |  |  |  |  |
| Energy intake (kcal/day) | | <1507 | | 670 (29.4) | | 293.3 (280.9,306.3) | | Ref. | | 190.1 (177.2,204.1) | | Ref. | | | 206.3 (193.6,219.9) | | Ref. | |  |  |  |  |
|  | | 1507-1905 | | 672 (29.4) | | 288.3 (276.1,301.1) | | -1.71 | | 190.6 (177.6,204.5) | | 0.23 | | | 209.0 (196.1,222.7) | | 1.28 | |  |  |  |  |
|  | | ≥1905 | | 672 (29.4) | | 288.7 (276.5,301.5) | | -1.58 | | 185.2 (172.6,198.7) | | -2.61 | | | 209.5 (196.6,223.3) | | 1.53 | |  |  |  |  |
| p-value, extreme categories | |  | |  | |  | | 0.610 | |  | | 0.603 | | |  | | 0.740 | |  |  |  |  |
| Waist-Hip-Ratio^c^ | | <0.80 | | 710 (31.1) | | 272.3 (260.8,284.2) | | Ref. | | 174.7 (163.0,187.3) | | Ref. | | | 193.0 (181.2,205.5) | | Ref. | |  |  |  |  |
|  | | 0.80-0.84 | | 711 (31.2) | | 284.5 (272.8,296.6) | | 4.49 | | 188.8 (176.4,202.0) | | 8.05 | | | 203.8 (191.7,216.8) | | 5.62 | |  |  |  |  |
|  | | ≥0.85 | | 861 (37.7) | | 307.2 (295.4,319.4) | | 12.83 | | 197.9 (185.7,210.8) | | 13.26 | | | 219.9 (207.6,232.9) | | 13.94 | |  |  |  |  |
| p-value, extreme categories | |  | |  | |  | | <.0001 | |  | | 0.011 | | |  | | 0.004 | |  |  |  |  |
| Study region | | Hamburg | | 1047 (45.9) | | 421.4 (407.0,436.3) | | Ref. | | 392.2 (370.9,414.8) | | Ref. | | | 443.4 (421.5,466.5) | | Ref. | |  |  |  |  |
|  | | Rhine-Neckar-Karlsruhe | | 1235 (54.1) | | 197.9 (191.7,204.3) | | -53.04 | | 89.7 (85.2,94.4) | | -77.14 | | | 95.8 (91.4,100.4) | | -78.40 | |  |  |  |  |
| p-value, extreme categories | |  | |  | |  | | <.0001 | |  | | <.0001 | | |  | | <.0001 | |  |  |  |  |

Geometric means (95% CI) calculated using generalized linear models adjusted for age at diagnosis (continuous; except exposure category age), BMI (continuous; except exposure category BMI), and study region (Hamburg, Rhine-Neckar-Karlsruhe; except exposure category study region).

Table S1 includes exposures not significantly associated with oxysterols or with ≤10% difference between exposure categories.

Significance level after Bonferroni correction p <0.0001.

^a^missing oxysterol values due to levels below LOD or levels exceeding calibration range; missing exposure categories values: alcohol consumption, n=2; vitamin D levels, n= 1179; total cholesterol intake, n=268; total energy intake, n=268.

^b^please note that these are not fixed subgroups but only created for a better overview

^c^WHR=waist-hip-ratio: <0.80=normal weight; 0.8-0.84=overweight; ≥0.85=obese. Results listed here are adjusted for BMI.

Abbreviations nM=nanomolar; LOD=level of detection; p-diff=percentage difference between extreme categories; WHR=waist-hip-ratio; 24-DHLan=24,25-dihydrolanosterol; 7-DC=7-dehydrocholesterol; 27-HC=27-hydroxycholesterol; 25-HC=25-hydroxycholesterol; 24S-HC=24S-hydroxycholesterol; 5a6a-EC=5α,6α-epoxycholesterol; 5b6b-EC=5β,6β-epoxycholesterol; THC=5α,6β-dihydroxycholestanol; 7a-HC=7α-hydroxycholesterol; 7b-HC=7β-hydroxycholesterol; 7-KC=7-ketocholesterol.

### Table S4: Additional cross-sectional associations between comorbidities and medication use at baseline and oxysterol concentrations by metabolic pathway

| **Exposure** | **Categories** | **n (%)^a^** | **Geometric mean  (95% CI), nM** | **% diff** | **Geometric mean  (95% CI), nM** | **% diff** | | |  | **Geometric mean  (95% CI), nM** | | **% diff** | | **Geometric mean  (95% CI), nM** | | **% diff** |
| --- | --- | --- | --- | --- | --- | --- | --- | --- | --- | --- | --- | --- | --- | --- | --- | --- |
| **Cholesterol precursors** | |  | **Lanosterol  (n=2282)** |  | **24-DHLan  (n=1027)** |  |  | **Desmosterol (n=2281)** | | | |  | | **7-DC (n=2221)** | |  |
| CCI | 0 | 1487 (65.2) | 544.8 (531.7,558.3) | Ref. | 40.4 (38.7,42.1) | Ref. | | | 1740.8 (1696.5,1786.2) | | Ref. | | 492.5 (465.3,521.3) | | Ref. | |
|  | 1 | 513 (22.5) | 513.4 (492.6,535.2) | -5.76 | 36.8 (34.1,39.7) | -8.84 | | | 1611.5 (1542.5,1683.7) | | -7.42 | | 513.2 (466.0,565.1) | | 4.20 | |
|  | 2 | 282 (12.4) | 515.9 (487.8,545.7) | -5.30 | 41.4 (37.6,45.6) | 2.56 | | | 1704.9 (1606.9,1808.8) | | -2.06 | | 483.8 (424.3,551.6) | | -1.77 | |
| p-value, extreme categories |  |  |  | 0.082 |  | 0.635 | | |  | | 0.529 | |  | | 0.808 | |
| Hypertension | No | 1338 (58.6) | 542.7 (528.6,557.2) | Ref. | 94.9 (93.2,96.6) | Ref. | | | 1866.2 (1816.0,1917.8) | | Ref. | | 443.9 (418.0,471.4) | | Ref. | |
|  | Yes | 941 (41.2) | 521.4 (505.1,538.2) | -3.92 | 93.7 (91.7,95.8) | -1.28 | | | 1500.5 (1452.0,1550.7) | | -19.59 | | 585.3 (543.2,630.6) | | 31.84 | |
| p-value, extreme categories |  |  |  | 0.064 |  | 0.073 | | |  | | <.0001 | |  | | <.0001 | |
| Osteoporosis | No | 1986 (87.0) | 534.0 (523.0,545.4) | Ref. | 94.2 (92.8,95.6) | Ref. | | | 1708.3 (1670.5,1746.9) | | Ref. | | 492.0 (468.5,516.8) | | Ref. | |
|  | Yes | 237 (10.4) | 540.2 (508.3,574.0) | 1.15 | 95.5 (91.6,99.7) | 1.43 | | | 1689.6 (1583.5,1802.7) | | -1.10 | | 537.9 (466.2,620.5) | | 9.32 | |
| p-value, extreme categories |  |  |  | 0.728 |  | 0.475 | | |  | | 0.753 | |  | | 0.248 | |
| Chronic lung disease | No | 2048 (89.7) | 537.1 (526.1,548.4) | Ref. | 94.6 (93.3,96.0) | Ref. | | | 1702.4 (1665.5,1740.1) | | Ref. | | 497.4 (473.9,521.9) | | Ref. | |
|  | Yes | 234 (10.3) | 507.4 (477.3,539.4) | -5.53 | 92.7 (88.9,96.7) | -2.03 | | | 1742.8 (1633.8,1859.2) | | 2.38 | | 484.2 (420.4,557.6) | | -2.65 | |
| p-value, extreme categories |  |  |  | 0.084 |  | 0.534 | | |  | | 0.499 | |  | | 0.724 | |
| **Cholesterol metabolites: enzymatic pathway^b^** | |  | **27-HC (n=2282)** |  | **25-HC (n=2282)** |  | | | **24S-HC (n=2279)** | |  | |  |  |  |  |
| CCI | 0 | 1487 (65.2) | 212.2 (209.6,214.9) | Ref. | 19.7 (19.1,20.3) | Ref. | | | 96.5 (94.9,98.1) | | Ref. | |  |  |  |  |
|  | 1 | 513 (22.5) | 207.2 (202.9,211.7) | -2.33 | 20.2 (19.1,21.2) | 2.42 | | | 90.3 (87.8,92.9) | | -6.37 | |  |  |  |  |
|  | 2 | 282 (12.4) | 198.5 (192.9,204.3) | -6.44 | 20.4 (19.0,21.9) | 3.83 | | | 91.3 (87.8,94.9) | | -5.37 | |  |  |  |  |
| p-value, extreme categories |  |  |  | <.0001 |  | 0.344 | | |  | | 0.011 | |  |  |  |  |
| Hypertension | No | 1338 (58.6) | 210.4 (207.5,213.2) | Ref. | 20.0 (19.3,20.6) | Ref. | | | 94.9 (93.2,96.6) | | Ref. | |  |  |  |  |
|  | Yes | 941 (41.2) | 207.8 (204.4,211.2) | -1.23 | 19.7 (19.0,20.5) | -1.17 | | | 93.7 (91.7,95.8) | | -1.28 | |  |  |  |  |
| p-value, extreme categories |  |  |  | 0.267 |  | 0.669 | | |  | | 0.389 | |  |  |  |  |
| Osteoporosis | No | 1986 (87.0) | 209.2 (207.0,211.5) | Ref. | 19.9 (19.4,20.5) | Ref. | | | 94.2 (92.8,95.6) | | Ref. | |  |  |  |  |
|  | Yes | 237 (10.4) | 209.6 (203.1,216.3) | 0.18 | 18.7 (17.3,20.2) | -6.15 | | | 95.5 (91.6,99.7) | | 1.43 | |  |  |  |  |
| p-value, extreme categories |  |  |  | 0.914 |  | 0.126 | | |  | | 0.534 | |  |  |  |  |
| Chronic lung disease | No | 2048 (89.7) | 210.1 (207.9,212.4) | Ref. | 19.9 (19.4,20.4) | Ref. | | | 94.6 (93.3,96.0) | | Ref. | |  |  |  |  |
|  | Yes | 234 (10.3) | 202.8 (196.5,209.3) | -3.47 | 19.9 (18.5,21.6) | 0.39 | | | 92.7 (88.9,96.7) | | -2.03 | |  |  |  |  |
| p-value, extreme categories |  |  |  | 0.037 |  | 0.926 | | |  | | 0.366 | |  |  |  |  |
| **Cholesterol metabolites: non- enzymatic pathway^b^** | |  | **5a6a-EC  (n=2281)** |  | **5b6b-EC  (n=2278)** |  | | | **THC (n=2282)** | |  | |  |  |  |  |
| CCI | 0 | 1487 (65.2) | 28.1 (27.2,28.9) | Ref. | 110.3 (107.2,113.6) | Ref. | | | 4.7 (4.4,5.0) | | Ref. | |  |  |  |  |
|  | 1 | 513 (22.5) | 28.1 (26.7,29.5) | 0.06 | 112.2 (106.8,117.9) | 1.72 | | | 4.3 (3.9,4.8) | | -8.36 | |  |  |  |  |
|  | 2 | 282 (12.4) | 27.3 (25.5,29.2) | -2.78 | 108.1 (101.1,115.5) | -2.06 | | | 4.7 (4.1,5.5) | | 0.15 | |  |  |  |  |
| p-value, extreme categories |  |  |  | 0.457 |  | 0.577 | | |  | | 0.986 | |  |  |  |  |
| Hypertension | No | 1338 (58.6) | 28.0 (27.1,28.9) | Ref. | 110.3 (106.9,113.8) | Ref. | | | 4.9 (4.5,5.2) | | Ref. | |  |  |  |  |
|  | Yes | 941 (41.2) | 27.9 (26.8,29.0) | -0.54 | 110.7 (106.6,115.0) | 0.41 | | | 4.3 (4.0,4.7) | | -11.30 | |  |  |  |  |
| p-value, extreme categories |  |  |  | 0.834 |  | 0.875 | | |  | | 0.041 | |  |  |  |  |
| Osteoporosis | No | 1986 (87.0) | 28.0 (27.3,28.7) | Ref. | 110.2 (107.5,113.0) | Ref. | | | 4.5 (4.3,4.8) | | Ref. | |  |  |  |  |
|  | Yes | 237 (10.4) | 27.4 (25.4,29.4) | -2.33 | 110.8 (103.1,119.1) | 0.55 | | | 5.4 (4.6,6.4) | | 19.93 | |  |  |  |  |
| p-value, extreme categories |  |  |  | 0.551 |  | 0.888 | | |  | | 0.042 | |  |  |  |  |
| Chronic lung disease | No | 2048 (89.7) | 27.9 (27.2,28.6) | Ref. | 110.2 (107.5,112.9) | Ref. | | | 4.6 (4.4,4.9) | | Ref. | |  |  |  |  |
|  | Yes | 234 (10.3) | 28.3 (26.3,30.5) | 1.32 | 113.3 (105.3,121.8) | 2.80 | | | 4.5 (3.8,5.3) | | -2.72 | |  |  |  |  |
| p-value, extreme categories |  |  |  | 0.741 |  | 0.482 | | |  | | 0.758 | |  |  |  |  |
| **Cholesterol metabolites: non- enzymatic pathway^b^** | |  | **7a-HC (n=2271)** |  | **7b-HC (n=2272)** |  | | | **7-KC (n=2282)** | |  | |  |  |  |  |
| CCI | 0 | 1487 (65.2) | 286.2 (278.0,294.7) | Ref. | 184.6 (176.1,193.5) | Ref. | | | 204.5 (195.9,213.5) | | Ref. | |  |  |  |  |
|  | 1 | 513 (22.5) | 293.8 (279.6,308.7) | 2.65 | 194.8 (179.8,211.0) | 5.55 | | | 213.7 (198.7,229.8) | | 4.48 | |  |  |  |  |
|  | 2 | 282 (12.4) | 293.0 (274.0,313.2) | 2.36 | 190.1 (170.7,211.8) | 3.02 | | | 201.0 (182.1,221.8) | | -1.74 | |  |  |  |  |
| p-value, extreme categories |  |  |  | 0.533 |  | 0.622 | | |  | | 0.749 | |  |  |  |  |
| Hypertension | No | 1338 (58.6) | 285.5 (276.7,294.6) | Ref. | 183.1 (174.1,192.7) | Ref. | | | 200.7 (191.7,210.2) | | Ref. | |  |  |  |  |
|  | Yes | 941 (41.2) | 293.2 (282.3,304.5) | 2.70 | 193.8 (182.2,206.0) | 5.79 | | | 214.0 (202.4,226.3) | | 6.63 | |  |  |  |  |
| p-value, extreme categories |  |  |  | 0.302 |  | 0.178 | | |  | | 0.091 | |  |  |  |  |
| Osteoporosis | No | 1986 (87.0) | 288.8 (281.6,296.2) | Ref. | 188.0 (180.5,195.7) | Ref. | | | 206.0 (198.5,213.7) | | Ref. | |  |  |  |  |
|  | Yes | 237 (10.4) | 291.6 (271.0,313.7) | 0.96 | 181.7 (161.6,204.4) | -3.31 | | | 199.4 (179.2,221.9) | | -3.18 | |  |  |  |  |
| p-value, extreme categories |  |  |  | 0.808 |  | 0.596 | | |  | | 0.575 | |  |  |  |  |
| Chronic lung disease | No | 2048 (89.7) | 287.8 (280.7,295.0) | Ref. | 184.6 (177.4,192.2) | Ref. | | | 205.7 (198.3,213.3) | | Ref. | |  |  |  |  |
|  | Yes | 234 (10.3) | 297.4 (276.5,319.9) | 3.36 | 214.7 (190.8,241.5) | 16.27 | | | 209.8 (188.5,233.6) | | 2.01 | |  |  |  |  |
| p-value, extreme categories |  |  |  | 0.399 |  | 0.017 | | |  | | 0.730 | |  |  |  |  |

Geometric means (95% CI) calculated using generalized linear models adjusted for age at diagnosis (continuous; except exposure category age), BMI (continuous; except exposure category BMI), and study region (Hamburg, Rhine-Neckar-Karlsruhe; except exposure category study region).

Table S2 includes exposures not significantly associated with oxysterols or with ≤10% difference between exposure categories.

Significance level after Bonferroni correction p <0.0001.

^a^missing oxysterol values due to levels below LOD or levels exceeding calibration range; missing exposure categories values: hypertension, n=3; osteoporosis, n=59

^b^please note that these are not fixed subgroups but only created for a better overview

Abbreviations: nM=nanomolar; LOD=level of detection; p-diff=percentage difference between extreme categories; CCI=Charlson Comorbidity Index; 24-DHLan=24,25-dihydrolanosterol; 7-DC=7-dehydrocholesterol; 27-HC=27-hydroxycholesterol; 25-HC=25-hydroxycholesterol; 24S-HC=24S-hydroxycholesterol; 5a6a-EC=5α,6α-epoxycholesterol; 5b6b-EC=5β,6β-epoxycholesterol; THC=5α,6β-dihydroxycholestanol; 7a-HC=7α-hydroxycholesterol; 7b-HC=7β-hydroxycholesterol; 7-KC=7-ketocholesterol.

### Table S5: Additional cross-sectional associations between breast cancer tumor and treatment characteristics and oxysterol concentrations by metabolic pathway

| **Exposure** | | **Categories** | | **n (%)^a^** | | **Geometric mean  (95% CI), nM** | | **% diff** | | **Geometric mean  (95% CI), nM** | | **% diff** | | | |  | **Geometric mean  (95% CI), nM** | | | **% diff** | | **Geometric mean  (95% CI), nM** | | | **% diff** |  |
| --- | --- | --- | --- | --- | --- | --- | --- | --- | --- | --- | --- | --- | --- | --- | --- | --- | --- | --- | --- | --- | --- | --- | --- | --- | --- | --- |
| **Cholesterol precursors** | | | |  | | **Lanosterol  (n=2282)** | |  | | **24-DHLan  (n=1027)** | |  |  | **Desmosterol (n=2281)** | | | | | |  | **7-DC (n=2221)** | | |  | | |
| Her2 | | HER2+ | | 444 (19.5) | | 561.0 (536.5,586.5) | | Ref. | | 41.4 (38.5,44.5) | | Ref. | | | | 1698.1 (1620.1,1779.8) | | Ref. | | 483.2 (435.3,536.3) | | | Ref. | | |  |
|  | | HER2- | | 1717 (75.2) | | 528.3 (516.4,540.4) | | -5.83 | | 39.4 (37.9,41.0) | | -4.69 | | | | 1711.3 (1670.8,1752.9) | | 0.78 | | 495.3 (469.6,522.3) | | | 2.5 | | |  |
| p-value, extreme categories | |  | |  | |  | | 0.019 | |  | | 0.252 | | | |  | | 0.773 | |  | | | 0.680 | | |  |
| Size | | <2cm | | 1401 (61.4) | | 520.2 (507.3,533.5) | | Ref. | | 38.5 (36.9,40.2) | | Ref. | | | | 1743.8 (1698.1,1790.7) | | Ref. | | 500.9 (472.4,531.0) | | | Ref. | | |  |
|  | | 2-5cm | | 807 (35.4) | | 554.5 (536.4,573.1) | | 6.58 | | 41.5 (39.2,44.0) | | 7.82 | | | | 1634.9 (1578.7,1693.1) | | -6.24 | | 485.7 (449.6,524.7) | | | -3.03 | | |  |
|  | | >5cm | | 72 (3.2) | | 583.3 (522.3,651.3) | | 12.11 | | 44.1 (36.8,52.8) | | 14.37 | | | | 1810.0 (1610.8,2033.9) | | 3.80 | | 527.2 (407.6,681.9) | | | 5.25 | | |  |
| p-value, extreme categories | |  | |  | |  | | 0.048 | |  | | 0.157 | | | |  | | 0.542 | |  | | | 0.704 | | |  |
| Grade | | Low | | 469 (20.6) | | 532.1 (509.6,555.5) | | Ref. | | 41.5 (38.5,44.8) | | Ref. | | | | 1785.0 (1705.0,1868.7) | | Ref. | | 501.8 (453.9,554.7) | | | Ref. | | |  |
|  | | Moderate | | 1234 (54.1) | | 527.2 (513.3,541.4) | | -0.92 | | 38.5 (36.8,40.3) | | -7.22 | | | | 1684.8 (1637.7,1733.2) | | -5.61 | | 496.6 (466.7,528.4) | | | -1.03 | | |  |
|  | | High | | 568 (24.9) | | 555.2 (533.9,577.4) | | 4.35 | | 40.9 (38.3,43.7) | | -1.41 | | | | 1694.8 (1625.9,1766.7) | | -5.05 | | 500.4 (457.1,547.9) | | | -0.27 | | |  |
| p-value, extreme categories | |  | |  | |  | | 0.153 | |  | | 0.783 | | | |  | | 0.101 | |  | | | 0.969 | | |  |
| Nodal Status (no. of positive lymph) | | 0 | | 1646 (72.1) | | 525.4 (513.4,537.6) | | Ref. | | 38.9 (37.4,40.5) | | Ref. | | | | 1733.3 (1691.5,1776.0) | | Ref. | | 485.2 (459.9,511.8) | | | Ref. | | |  |
|  | | 1-3 | | 510 (22.3) | | 550.3 (528.0,573.6) | | 4.75 | | 40.4 (37.6,43.3) | | 3.70 | | | | 1616.1 (1546.9,1688.4) | | -6.76 | | 531.2 (481.8,585.6) | | | 9.48 | | |  |
|  | | 4-9 | | 126 (5.5) | | 584.9 (538.1,635.7) | | 11.33 | | 49.0 (42.4,56.6) | | 25.77 | | | | 1735.1 (1589.0,1894.7) | | 0.11 | | 505.5 (416.8,613.0) | | | 4.19 | | |  |
| p-value, extreme categories | |  | |  | |  | | 0.015 | |  | | 0.003 | | | |  | | 0.982 | |  | | | 0.688 | | |  |
| Chemotherapy | | No | | 1218 (53.4) | | 510.4 (496.7,524.5) | | Ref. | | 38.1 (36.3,40.0) | | Ref. | | | | 1719.1 (1669.8,1769.7) | | Ref. | | 488.2 (458.2,520.3) | | | Ref. | | |  |
|  | | Yes | | 1040 (45.6) | | 563.1 (546.8,579.9) | | 10.33 | | 41.6 (39.6,43.7) | | 9.04 | | | | 1693.4 (1641.3,1747.1) | | -1.49 | | 503.2 (469.8,538.9) | | | 3.06 | | |  |
| p-value, extreme categories | |  | |  | |  | | <.0001 | |  | | 0.014 | | | |  | | 0.495 | |  | | | 0.533 | | |  |
| Radiotherapy | | No | | 435 (19.1) | | 535.6 (512.0,560.3) | | Ref. | | 38.0 (35.3,40.8) | | Ref. | | | | 1667.5 (1590.1,1748.7) | | Ref. | | 472.7 (425.9,524.7) | | | Ref. | | |  |
|  | | Yes | | 1828 (80.1) | | 534.4 (522.8,546.2) | | -0.23 | | 40.1 (38.6,41.7) | | 5.75 | | | | 1720.3 (1680.9,1760.7) | | 3.17 | | 500.5 (475.6,526.6) | | | 5.87 | | |  |
| p-value, extreme categories | |  | |  | |  | | 0.929 | |  | | 0.184 | | | |  | | 0.248 | |  | | | 0.335 | | |  |
| Endocrine therapy^c^ (blood collection ≥ 3 months after diagnosis) | | None | | 173 (14.5) | | 589.8 (549.3,633.2) | | Ref. | | 43.3 (38.3,48.9) | | Ref. | | | | 1724.2 (1600.4,1857.6) | | Ref. | | 506.7 (426.8,601.5) | | | Ref. | | |  |
|  | | Tam + AI | | 389 (32.6) | | 525.0 (500.6,550.6) | | -10.98 | | 35.2 (32.2,38.4) | | -18.73 | | | | 1804.1 (1716.3,1896.4) | | 4.64 | | 555.8 (495.7,623.2) | | | 9.7 | | |  |
|  | | Tam only | | 467 (39.1) | | 490.0 (468.8,512.2) | | -16.92 | | 33.6 (30.7,36.9) | | -22.25 | | | | 1816.0 (1733.6,1902.3) | | 5.32 | | 508.8 (457.5,565.8) | | | 0.42 | | |  |
|  | | AI only | | 124 (10.4) | | 575.5 (528.6,626.6) | | -2.42 | | 48.0 (40.7,56.6) | | 10.87 | | | | 1694.3 (1549.8,1852.2) | | -1.73 | | 476.1 (386.8,585.9) | | | -6.04 | | |  |
|  | | Unknown | | 40 (3.4) | | 568.7 (490.5,659.3) | | -3.58 | | 40.8 (30.4,54.6) | | -5.8 | | | | 1968.1 (1685.7,2297.7) | | 14.15 | | 589.1 (414.8,836.6) | | | 16.27 | | |  |
| p-value, extreme categories | |  | |  | |  | | 0.008 | |  | | 0.007 | | | |  | | 0.321 | |  | | | 0.379 | | |  |
| **Cholesterol metabolites: enzymatic pathway^b^** | | | |  | | **27-HC (n=2282)** | |  | | **25-HC (n=2282)** | |  | | | | **24S-HC (n=2279)** | |  | |  |  |  |  |  |  |  |
| Her2 | | HER2+ | | 444 (19.5) | | 211.6 (206.8,216.5) | | Ref. | | 19.7 (18.6,20.8) | | Ref. | | | | 96.6 (93.6,99.6) | | Ref. | |  |  |  |  |  |  |  |
|  | | HER2- | | 1717 (75.2) | | 209.0 (206.5,211.4) | | -1.26 | | 19.8 (19.3,20.4) | | 0.7 | | | | 93.9 (92.5,95.4) | | -2.72 | |  |  |  |  |  |  |  |
| p-value, extreme categories | |  | |  | |  | | 0.333 | |  | | 0.831 | | | |  | | 0.118 | |  |  |  |  |  |  |  |
| Grade | | Low | | 469 (20.6) | | 211.0 (206.4,215.8) | | Ref. | | 19.6 (18.5,20.7) | | Ref. | | | | 92.8 (90.1,95.6) | | Ref. | |  |  |  |  |  |  |  |
|  | | Moderate | | 1234 (54.1) | | 206.5 (203.7,209.4) | | -2.13 | | 19.6 (19.0,20.3) | | 0.21 | | | | 94.0 (92.3,95.8) | | 1.27 | |  |  |  |  |  |  |  |
|  | | High | | 568 (24.9) | | 214.7 (210.5,219.1) | | 1.76 | | 20.6 (19.6,21.7) | | 5.36 | | | | 96.9 (94.4,99.6) | | 4.44 | |  |  |  |  |  |  |  |
| p-value, extreme categories | |  | |  | |  | | 0.253 | |  | | 0.168 | | | |  | | 0.035 | |  |  |  |  |  |  |  |
| Size | | <2cm | | 1401 (61.4) | | 208.4 (205.8,211.2) | | Ref. | | 19.4 (18.8,20.0) | | Ref. | | | | 93.7 (92.1,95.3) | | Ref. | |  |  |  |  |  |  |  |
|  | | 2-5cm | | 807 (35.4) | | 211.2 (207.6,214.8) | | 1.31 | | 20.7 (19.9,21.6) | | 6.75 | | | | 96.1 (93.9,98.3) | | 2.58 | |  |  |  |  |  |  |  |
|  | | >5cm | | 72 (3.2) | | 207.0 (195.6,219.1) | | -0.69 | | 20.7 (18.0,23.8) | | 6.61 | | | | 90.4 (83.7,97.5) | | -3.55 | |  |  |  |  |  |  |  |
| p-value, extreme categories | |  | |  | |  | | 0.817 | |  | | 0.382 | | | |  | | 0.365 | |  |  |  |  |  |  |  |
| Nodal Status (no. of positive lymph) | | 0 | | 1646 (72.1) | | 208.9 (206.4,211.4) | | Ref. | | 19.8 (19.2,20.4) | | Ref. | | | | 93.8 (92.4,95.4) | | Ref. | |  |  |  |  |  |  |  |
|  | | 1-3 | | 510 (22.3) | | 210.0 (205.6,214.6) | | 0.54 | | 19.7 (18.7,20.8) | | -0.27 | | | | 94.7 (92.0,97.5) | | 0.93 | |  |  |  |  |  |  |  |
|  | | 4-9 | | 126 (5.5) | | 212.5 (203.6,221.9) | | 1.75 | | 22.0 (19.8,24.4) | | 11.19 | | | | 100.8 (95.2,106.8) | | 7.40 | |  |  |  |  |  |  |  |
| p-value, extreme categories | |  | |  | |  | | 0.446 | |  | | 0.058 | | | |  | | 0.019 | |  |  |  |  |  |  |  |
| Chemo | | No | | 1218 (53.4) | | 206.7 (203.8,209.6) | | Ref. | | 19.4 (18.7,20.1) | | Ref. | | | | 92.4 (90.6,94.1) | | Ref. | |  |  |  |  |  |  |  |
|  | | Yes | | 1040 (45.6) | | 212.5 (209.4,215.8) | | 2.82 | | 20.4 (19.6,21.2) | | 5.12 | | | | 96.7 (94.8,98.7) | | 4.73 | |  |  |  |  |  |  |  |
| p-value, extreme categories | |  | |  | |  | | 0.009 | |  | | 0.059 | | | |  | | 0.001 | |  |  |  |  |  |  |  |
| Radiotherapy | | No | | 435 (19.1) | | 205.6 (200.9,210.4) | | Ref. | | 19.5 (18.4,20.6) | | Ref. | | | | 92.6 (89.8,95.6) | | Ref. | |  |  |  |  |  |  |  |
|  | | Yes | | 1828 (80.1) | | 210.1 (207.7,212.5) | | 2.16 | | 19.9 (19.4,20.5) | | 2.43 | | | | 94.8 (93.3,96.2) | | 2.30 | |  |  |  |  |  |  |  |
| p-value, extreme categories | |  | |  | |  | | 0.104 | |  | | 0.460 | | | |  | | 0.197 | |  |  |  |  |  |  |  |
| Endocrine therapy^c^ (blood collection ≥ 3 months after diagnosis) | None | | 173 (14.5) | | 213.6 (206.1,221.3) | | Ref. | | 19.5 (17.8,21.5) | | Ref. | | | | 95.5 (91.0,100.1) | | | Ref. |  |  |  |  |  |  |  |  |
|  | Tam + AI | | 389 (32.6) | | 202.7 (197.9,207.6) | | -5.1 | | 18.7 (17.6,19.9) | | -4.23 | | | | 92.6 (89.7,95.6) | | | -2.99 |  |  |  |  |  |  |  |  |
|  | Tam only | | 467 (39.1) | | 197.8 (193.5,202.2) | | -7.38 | | 18.6 (17.5,19.7) | | -4.85 | | | | 88.4 (85.8,91.0) | | | -7.44 |  |  |  |  |  |  |  |  |
|  | AI only | | 124 (10.4) | | 211.7 (202.9,220.9) | | -0.86 | | 18.5 (16.5,20.8) | | -5.18 | | | | 94.5 (89.3,100.1) | | | -0.99 |  |  |  |  |  |  |  |  |
|  | Unknown | | 40 (3.4) | | 223.8 (207.9,241.0) | | 4.81 | | 18.3 (15.1,22.3) | | -6.12 | | | | 101.3 (91.8,111.9) | | | 6.13 |  |  |  |  |  |  |  |  |
| p-value, extreme categories |  | |  | |  | | 0.379 | |  | | 0.457 | | | |  | | | 0.300 |  |  |  |  |  |  |  |  |
| **Cholesterol metabolites: non- enzymatic pathway^b^** | | | |  | | **5a6a-EC  (n=2281)** | |  | | **5b6b-EC  (n=2278)** | |  | | | | **THC (n=2282)** | |  | |  |  |  |  |  |  |  |
| Her2 | | HER2+ | | 444 (19.5) | | 27.8 (26.3,29.3) | | Ref. | | 110.7 (105.0,116.7) | | Ref. | | | | 4.9 (4.4,5.6) | | Ref. | |  |  |  |  |  |  |  |
|  | | HER2- | | 1717 (75.2) | | 27.9 (27.1,28.7) | | 0.47 | | 109.9 (107.0,112.9) | | -0.73 | | | | 4.6 (4.3,4.8) | | -7.67 | |  |  |  |  |  |  |  |
| p-value, extreme categories | |  | |  | |  | | 0.880 | |  | | 0.809 | | | |  | | 0.249 | |  |  |  |  |  |  |  |
| Grade | | Low | | 469 (20.6) | | 28.8 (27.4,30.4) | | Ref. | | 113.4 (107.6,119.4) | | Ref. | | | | 4.4 (3.9,4.9) | | Ref. | |  |  |  |  |  |  |  |
|  | | Moderate | | 1234 (54.1) | | 27.7 (26.8,28.6) | | -4.01 | | 110.1 (106.6,113.7) | | -2.87 | | | | 4.6 (4.3,5.0) | | 5.41 | |  |  |  |  |  |  |  |
|  | | High | | 568 (24.9) | | 27.8 (26.5,29.2) | | -3.53 | | 108.9 (103.9,114.2) | | -3.92 | | | | 4.9 (4.4,5.5) | | 11.86 | |  |  |  |  |  |  |  |
| p-value, extreme categories | |  | |  | |  | | 0.319 | |  | | 0.263 | | | |  | | 0.167 | |  |  |  |  |  |  |  |
| Size | | <2cm | | 1401 (61.4) | | 27.6 (26.8,28.4) | | Ref. | | 109.5 (106.2,112.8) | | Ref. | | | | 4.3 (4.0,4.6) | | Ref. | |  |  |  |  |  |  |  |
|  | | 2-5cm | | 807 (35.4) | | 28.6 (27.5,29.8) | | 3.84 | | 112.3 (107.9,116.8) | | 2.60 | | | | 5.3 (4.8,5.8) | | 23.91 | |  |  |  |  |  |  |  |
|  | | >5cm | | 72 (3.2) | | 28.0 (24.5,32.0) | | 1.64 | | 110.4 (96.8,126.0) | | 0.91 | | | | 5.1 (3.8,6.9) | | 20.56 | |  |  |  |  |  |  |  |
| p-value, extreme categories | |  | |  | |  | | 0.816 | |  | | 0.896 | | | |  | | 0.233 | |  |  |  |  |  |  |  |
| Nodal Status (no. of positive lymph) | | 0 | | 1646 (72.1) | | 27.7 (27.0,28.5) | | Ref. | | 109.6 (106.6,112.7) | | Ref. | | | | 4.4 (4.1,4.7) | | Ref. | |  |  |  |  |  |  |  |
|  | | 1-3 | | 510 (22.3) | | 28.2 (26.8,29.6) | | 1.59 | | 110.7 (105.3,116.3) | | 0.97 | | | | 5.2 (4.6,5.8) | | 18.02 | |  |  |  |  |  |  |  |
|  | | 4-9 | | 126 (5.5) | | 30.3 (27.4,33.5) | | 9.34 | | 121.5 (110.0,134.2) | | 10.85 | | | | 6.0 (4.7,7.5) | | 35.57 | |  |  |  |  |  |  |  |
| p-value, extreme categories | |  | |  | |  | | 0.093 | |  | | 0.051 | | | |  | | 0.011 | |  |  |  |  |  |  |  |
| Chemotherapy | | No | | 1218 (53.4) | | 27.6 (26.7,28.6) | | Ref. | | 109.7 (106.2,113.3) | | Ref. | | | | 4.1 (3.8,4.4) | | Ref. | |  |  |  |  |  |  |  |
|  | | Yes | | 1040 (45.6) | | 28.3 (27.3,29.4) | | 2.56 | | 111.3 (107.4,115.2) | | 1.42 | | | | 5.3 (4.9,5.8) | | 29.21 | |  |  |  |  |  |  |  |
| p-value, extreme categories | |  | |  | |  | | 0.316 | |  | | 0.571 | | | |  | | <.0001 | |  |  |  |  |  |  |  |
| Radiotherapy | | No | | 435 (19.1) | | 27.5 (26.1,29.1) | | Ref. | | 107.9 (102.2,113.8) | | Ref. | | | | 4.7 (4.1,5.3) | | Ref. | |  |  |  |  |  |  |  |
|  | | Yes | | 1828 (80.1) | | 28.1 (27.3,28.8) | | 1.98 | | 111.0 (108.2,114.0) | | 2.96 | | | | 4.6 (4.4,4.9) | | -0.75 | |  |  |  |  |  |  |  |
| p-value, extreme categories | |  | |  | |  | | 0.524 | |  | | 0.340 | | | |  | | 0.914 | |  |  |  |  |  |  |  |
| Endocrine therapy^c^ (blood collection ≥ 3 months after diagnosis) | | None | | 173 (14.5) | | 28.7 (26.3,31.4) | | Ref. | | 108.9 (99.8,118.8) | | Ref. | | | | 5.7 (4.7,6.9) | | Ref. | |  |  |  |  |  |  |  |
|  | | Tam + AI | | 389 (32.6) | | 28.2 (26.6,29.9) | | -1.87 | | 111.2 (105.0,117.9) | | 2.15 | | | | 4.0 (3.6,4.6) | | -28.61 | |  |  |  |  |  |  |  |
|  | | Tam only | | 467 (39.1) | | 27.6 (26.1,29.1) | | -4.05 | | 108.2 (102.5,114.2) | | -0.63 | | | | 4.0 (3.6,4.5) | | -28.92 | |  |  |  |  |  |  |  |
|  | | AI only | | 124 (10.4) | | 27.1 (24.3,30.1) | | -5.8 | | 102.5 (92.4,113.7) | | -5.9 | | | | 4.5 (3.6,5.7) | | -20.11 | |  |  |  |  |  |  |  |
|  | | Unkown | | 40 (3.4) | | 25.9 (21.6,31.1) | | -9.81 | | 104.1 (86.9,124.7) | | -4.44 | | | | 4.3 (2.9,6.5) | | -23.31 | |  |  |  |  |  |  |  |
| p-value, extreme categories | |  | |  | |  | | 0.727 | |  | | 0.690 | | | |  | | 0.004 | |  |  |  |  |  |  |  |
| **Cholesterol metabolites: non- enzymatic pathway^b^** | | | |  | | **7a-HC (n=2271)** | |  | | **7b-HC (n=2272)** | |  | | | | **7-KC (n=2282)** | |  | |  |  |  |  |  |  |  |
| Her2 | | HER2+ | | 444 (19.5) | | 284.4 (269.7,299.9) | | Ref. | | 181.1 (166.1,197.6) | | Ref. | | | | 209.0 (193.3,226.0) | | Ref. | |  |  |  |  |  |  |  |
|  | | HER2- | | 1717 (75.2) | | 289.3 (281.6,297.2) | | 1.72 | | 187.6 (179.5,196.1) | | 3.57 | | | | 203.9 (195.9,212.2) | | -2.46 | |  |  |  |  |  |  |  |
| p-value, extreme categories | |  | |  | |  | | 0.574 | |  | | 0.479 | | | |  | | 0.578 | |  |  |  |  |  |  |  |
| Size | | <2cm | | 1401 (61.4) | | 291.1 (282.5,300.0) | | Ref. | | 188.1 (179.2,197.5) | | Ref. | | | | 204.6 (195.8,213.9) | | Ref. | |  |  |  |  |  |  |  |
|  | | 2-5cm | | 807 (35.4) | | 286.1 (275.0,297.7) | | -1.72 | | 187.3 (175.6,199.7) | | -0.45 | | | | 209.3 (197.4,221.8) | | 2.27 | |  |  |  |  |  |  |  |
|  | | >5cm | | 72 (3.2) | | 276.8 (242.6,315.7) | | -4.93 | | 180.7 (146.0,223.6) | | -3.96 | | | | 201.7 (166.1,244.8) | | -1.46 | |  |  |  |  |  |  |  |
| p-value, extreme categories | |  | |  | |  | | 0.464 | |  | | 0.717 | | | |  | | 0.885 | |  |  |  |  |  |  |  |
| Grade | | Low | | 469 (20.6) | | 305.5 (290.1,321.6) | | Ref. | | 196.1 (180.4,213.2) | | Ref. | | | | 212.4 (196.8,229.1) | | Ref. | |  |  |  |  |  |  |  |
|  | | Moderate | | 1234 (54.1) | | 290.8 (281.7,300.2) | | -4.81 | | 190.6 (181.0,200.7) | | -2.82 | | | | 206.9 (197.4,216.8) | | -2.58 | |  |  |  |  |  |  |  |
|  | | High | | 568 (24.9) | | 271.4 (258.9,284.4) | | -11.16 | | 173.9 (161.2,187.6) | | -11.35 | | | | 198.8 (185.5,213.0) | | -6.40 | |  |  |  |  |  |  |  |
| p-value, extreme categories | |  | |  | |  | | 0.0009 | |  | | 0.037 | | | |  | | 0.208 | |  |  |  |  |  |  |  |
| Nodal Status (no. of positive lymph) | | 0 | | 1646 (72.1) | | 291.1 (283.2,299.2) | | Ref. | | 188.7 (180.4,197.3) | | Ref. | | | | 205.1 (197.0,213.6) | | Ref. | |  |  |  |  |  |  |  |
|  | | 1-3 | | 510 (22.3) | | 284.0 (270.2,298.4) | | -2.45 | | 183.6 (169.5,198.9) | | -2.70 | | | | 205.5 (191.1,221.0) | | 0.17 | |  |  |  |  |  |  |  |
|  | | 4-9 | | 126 (5.5) | | 278.0 (251.7,307.1) | | -4.49 | | 188.4 (160.5,221.2) | | -0.15 | | | | 221.9 (191.7,256.8) | | 8.16 | |  |  |  |  |  |  |  |
| p-value, extreme categories | |  | |  | |  | | 0.383 | |  | | 0.986 | | | |  | | 0.311 | |  |  |  |  |  |  |  |
| Chemotherapy | | No | | 1218 (53.4) | | 294.2 (284.7,303.9) | | Ref. | | 191.6 (181.7,202.0) | | Ref. | | | | 206.9 (197.2,217.1) | | Ref. | |  |  |  |  |  |  |  |
|  | | Yes | | 1040 (45.6) | | 282.5 (272.8,292.7) | | -3.95 | | 182.8 (172.7,193.5) | | -4.57 | | | | 205.3 (195.0,216.2) | | -0.77 | |  |  |  |  |  |  |  |
| p-value, extreme categories | |  | |  | |  | | 0.105 | |  | | 0.245 | | | |  | | 0.833 | |  |  |  |  |  |  |  |
| Radiotherapy | | No | | 435 (19.1) | | 272.9 (258.7,288.0) | | Ref. | | 177.9 (163.1,194.1) | | Ref. | | | | 200.3 (185.1,216.7) | | Ref. | |  |  |  |  |  |  |  |
|  | | Yes | | 1828 (80.1) | | 292.6 (285.1,300.4) | | 7.21 | | 189.8 (181.9,198.0) | | 6.69 | | | | 207.4 (199.6,215.6) | | 3.56 | |  |  |  |  |  |  |  |
| p-value, extreme categories | |  | |  | |  | | 0.022 | |  | | 0.189 | | | |  | | 0.434 | |  |  |  |  |  |  |  |
| Endocrine therapy^c^ (blood collection ≥ 3 months after diagnosis) | | None | | 173 (14.5) | | 279.3 (256.0,304.7) | | Ref. | | 169.0 (147.4,193.7) | | Ref. | | | | 204.8 (180.2,232.7) | | Ref. | |  |  |  |  |  |  |  |
|  | | Tam + AI | | 389 (32.6) | | 318.7 (300.7,337.7) | | 14.11 | | 196.3 (179.3,215.0) | | 16.19 | | | | 224.8 (206.4,244.9) | | 9.78 | |  |  |  |  |  |  |  |
|  | | Tam only | | 467 (39.1) | | 309.9 (293.6,327.0) | | 10.95 | | 204.4 (187.9,222.4) | | 20.96 | | | | 205.4 (189.7,222.4) | | 0.3 | |  |  |  |  |  |  |  |
|  | | AI only | | 124 (10.4) | | 286.3 (258.2,317.5) | | 2.52 | | 171.4 (145.8,201.6) | | 1.45 | | | | 176.4 (151.4,205.5) | | -13.85 | |  |  |  |  |  |  |  |
|  | | Unknown | | 40 (3.4) | | 260.6 (217.7,312.0) | | -6.68 | | 181.5 (137.0,240.5) | | 7.41 | | | | 174.9 (134.1,228.0) | | -14.61 | |  |  |  |  |  |  |  |
| p-value, extreme categories | |  | |  | |  | | 0.014 | |  | | 0.073 | | | |  | | 0.233 | |  |  |  |  |  |  |  |

Geometric means (95% CI) calculated using generalized linear models adjusted for age at diagnosis (continuous; except exposure category age), BMI (continuous; except exposure category BMI), and study region (Hamburg, Rhine-Neckar-Karlsruhe; except exposure category study region).

Table S3 includes exposures not significantly associated with oxysterols or with ≤10% difference between exposure categories.

Significance level after Bonferroni correction p <0.0001.

^a^missing oxysterol values due to levels below LOD or levels exceeding calibration range; missing exposure categories values: Her2-status, n=121; ; tumor size, n=2; chemotherapy, n=24; radiotherapy, n=19; time between OP and blood draw, n=780.

^b^please note that these are not fixed subgroups but only created for a better overview

^c^endocrine therapy among participants whose blood was drawn ≥ 3 months after the breast cancer diagnosis (n=1193): None=neither tamoxifen nor AI; Tam+AI=tamoxifen and aromatase inhibitors; Tam only=tamoxifen (no aromatase inhibitors); AI only = aromatase inhibitors (no tamoxifen).

Abbreviations: nM=nanomolar; LOD=level of detection; p-diff=percentage difference between extreme categories; 24-DHLan=24,25-dihydrolanosterol; 7-DC=7-dehydrocholesterol; 27-HC=27-hydroxycholesterol; 25-HC=25-hydroxycholesterol; 24S-HC=24S-hydroxycholesterol; 5a6a-EC=5α,6α-epoxycholesterol; 5b6b-EC=5β,6β-epoxycholesterol; THC=5α,6β-dihydroxycholestanol; 7a-HC=7α-hydroxycholesterol; 7b-HC=7β-hydroxycholesterol; 7-KC=7-ketocholesterol.

### Table S6: Cross-sectional associations between breast cancer tumor and treatment characteristics and oxysterol concentrations, mutually adjusted for exposures strongly associated with oxysterols (>10% difference and p<0.05) in addition to age, BMI, center

| **Effect** | **Categories** | **N (%)** | **Geometric mean (95% CI)** | **%diff** |
| --- | --- | --- | --- | --- |
|  |  |  |  | |
| **Lanosterol** |  |  |  | |
| *Mutually adjusted for age, BMI, statin use, breast cancer stage, chemotherapy* | | | | |
| Age at diagnosis (years) | < 55 | 332 (14.5) | 482.3 (451.9,514.8) | Ref. |
|  | 55 - 59 | 452 (19.8) | 501.1 (472.9,531.1) | 3.90 |
|  | 60 - 64 | 675 (29.6) | 506.4 (481.2,533.0) | 5.00 |
|  | 65 - 69 | 566 (24.8) | 498.8 (473.8,525.2) | 3.42 |
|  | 70 + | 257 (11.3) | 463.7 (434.1,495.3) | -3.86 |
| BMI (kg/m^2^) | < 18.5 | 35 (1.5) | 475.4 (404.7,558.5) | -2.06 |
|  | 18.5 -24.9 | 1031 (45.2) | 485.4 (462.4,509.6) | Ref. |
|  | 25-29.9 | 847 (37.1) | 495.5 (472.5,519.7) | 2.08 |
|  | >30 | 368 (16.1) | 532.3 (503.1,563.2) | 9.65* |
| Study region | Hamburg | 1047 (45.9) | 474.6 (453.1,497.2) | Ref |
|  | Rhine-Neckar-Karlsruhe | 1235 (54.1) | 514.1 (491.9,537.3) | 8.31** |
| Diabetes | No | 2070 (90.7) | 526.7 (510.9,543.0) | Ref |
|  | Yes | 207 (9.1) | 463.3 (432.3,496.5) | -12.04* |
| Statin use | No | 1378 (60.4) | 539.1 (516.2,562.9) | Ref |
|  | Yes | 300 (13.1) | 436.7 (409.8,465.4) | -18.99** |
|  | Unknown | 601 (26.3) | 512.0 (487.5,537.7) | -5.02 |
| Stage | I | 1137 (49.8) | 475.2 (455.0,496.3) | Ref |
|  | IIa | 718 (31.5) | 493.7 (471.6,516.9) | 3.90 |
|  | IIb | 273 (12.0) | 486.9 (455.8,520.2) | 2.47 |
|  | IIIa | 154 (6.7) | 521.2 (479.7,566.2) | 9.68* |
| Chemo | No | 1218 (53.4) | 474.0 (450.5,498.7) | Ref |
|  | Yes | 1040 (45.6) | 514.7 (493.0,537.4) | 8.59* |
| **Desmosterol** |  |  |  |  |
| *Mutually adjusted for age, BMI, study region, physical activity, CVD, diabetes, statin use (except for aspirin), aspirin use (except for statin use), tumor stage, time between diagnosis and blood collection* | | | | |
| Age at diagnosis (years) | < 55 | 331 (14.5) | 1498.7 (1401.1,1603.2) | Ref. |
|  | 55 - 59 | 452 (19.8) | 1552.4 (1460.8,1649.7) | 3.58 |
|  | 60 - 64 | 675 (29.6) | 1503.5 (1423.2,1588.3) | 0.32 |
|  | 65 - 69 | 566 (24.8) | 1452.5 (1374.5,1535.1) | -3.08 |
|  | 70 + | 257 (11.3) | 1474.0 (1373.9,1581.3) | -1.65 |
| BMI (kg/m2) | < 18.5 | 35 (1.5) | 1461.2 (1235.6,1728.1) | -2.23 |
|  | 18.5 -24.9 | 1031 (45.2) | 1494.6 (1419.3,1573.9) | Ref. |
|  | 25-29.9 | 847 (37.1) | 1494.5 (1419.7,1573.3) | -0.01 |
|  | >30 | 368 (16.1) | 1509.6 (1420.9,1603.7) | 1 |
| Study region | Hamburg | 1046 (45.9) | 1448.6 (1377.5,1523.4) | Ref. |
|  | Rhine-Neckar-Karlsruhe | 1235 (54.1) | 1544.4 (1473.2,1619.1) | 6.62* |
| CVD | No | 1136 (49.8) | 1604.6 (1522.2,1691.4) | Ref. |
|  | Yes | 1145 (50.2) | 1386.8 (1323.3,1453.3) | -13.57** |
| Diabetes | No | 2069 (90.7) | 1612.0 (1557.1,1668.9) | Ref. |
|  | Yes | 207 (9.1) | 1387.9 (1290.3,1492.8) | -13.9** |
| Leisure physical activity (met*h/week) | < 28 |  | 1438.1 (1363.1,1517.2) | Ref. |
|  | ≥28 |  | 1555.7 (1486.6,1628.0) | 8.17* |
| Statin use | No | 1378 (60.4) | 1609.2 (1537.8,1684.0) | Ref. |
|  | Yes | 300 (13.2) | 1368.8 (1280.4,1463.3) | -14.94** |
|  | Unknown | 600 (26.3) | 1546.6 (1470.1,1627.1) | -3.89 |
| Aspirin | No | 1416 (62.1) | 1582.1 (1511.2,1656.2) | Ref. |
|  | Yes | 265 (11.6) | 1474.4 (1375.9,1580.0) | -6.81* |
|  | Unknown | 600 (26.3) | 1549.1 (1472.2,1630.0) | -2.08 |
| Stage | I | 1136 (49.8) | 1548.8 (1479.0,1621.9) | Ref. |
|  | IIa | 718 (31.5) | 1476.3 (1405.0,1551.2) | -4.68* |
|  | IIb | 273 (12.0) | 1411.8 (1319.0,1511.1) | -8.85* |
|  | IIIa | 154 (6.8) | 1550.5 (1424.4,1687.8) | 0.11 |
| Time between diagnosis and blood draw | ≤ 3 months | 1089 (47.7) | 1428.8 (1361.4,1499.5) | Ref. |
|  | >3 months | 1192 (52.3) | 1565.8 (1491.0,1644.3) | 9.59** |
| **7-DC** |  |  |  |  |
| *Mutually adjusted for age, BMI, study region, parity, CVD, statin use, tumor stage, endocrine therapy, time between diagnosis and blood draw* | | | | |
| Age at diagnosis (years) | < 55 | 326 (14.7) | 461.6 (368.7,577.8) | Ref. |
|  | 55 - 59 | 443 (19.9) | 412.0 (333.4,509.0) | -10.75 |
|  | 60 - 64 | 656 (29.5) | 497.5 (404.8,611.6) | 7.79 |
|  | 65 - 69 | 545 (24.5) | 504.5 (410.5,620.1) | 9.31 |
|  | 70 + | 251 (11.3) | 454.6 (363.0,569.2) | -1.52 |
| BMI (kg/m^2^) | < 18.5 | 34 (1.5) | 481.3 (318.1,728.2) | Ref. |
|  | 18.5 -24.9 | 1014 (45.7) | 422.5 (345.7,516.4) | 13.93 |
|  | 25-29.9 | 824 (37.1) | 489.1 (400.1,597.9) | 15.76* |
|  | >30 | 349 (15.7) | 584.8 (473.7,721.9) | 38.42** |
| Study region | Hamburg | 1022 (46.0) | 390.0 (319.5,476.1) | Ref. |
|  | Rhine-Neckar-Karlsruhe | 1199 (54.0) | 548.6 (451.6,666.4) | 40.64** |
| Parity | 0 | 382 (16.7) | 455.5 (399.8,518.9) | Ref. |
|  | 1 | 643 (28.2) | 528.1 (474.5,587.7) | 15.95* |
|  | 2+ | 1257 (55.1) | 500.6 (457.1,548.3) | 9.92 |
| CVD | No | 1132 (51.0) | 437.9 (396.8,483.3) | Ref. |
|  | Yes | 1089 (49.0) | 556.8 (506.2,612.5) | 27.14** |
| Statin use | No | 1345 (60.6) | 536.1 (488.1,588.8) | Ref. |
|  | Yes | 288 (13.0) | 475.8 (410.9,551.0) | -11.24* |
|  | Unknown | 585 (26.3) | 472.1 (426.4,522.7) | . |
| Stage | I | 1119 (50.4) | 477.6 (437.2,521.7) | Ref. |
|  | IIa | 688 (31.0) | 458.0 (415.0,505.5) | -4.09 |
|  | IIb | 263 (11.8) | 534.3 (461.9,617.9) | 11.87 |
|  | IIIa | 151 (6.8) | 508.8 (423.7,611.1) | 6.54 |
| Endocrine therapy | None | 343 (15.4) | 459.0 (404.0,521.4) | Ref. |
|  | Tam + AI | 715 (32.2) | 516.5 (468.1,569.8) | 12.53 |
|  | Tam only | 839 (37.8) | 482.0 (437.1,531.6) | 5.03 |
|  | AI only | 245 (11.0) | 434.7 (375.9,502.8) | -5.28 |
|  | Unknown | 79 (3.6) | 591.2 (456.5,765.6) | 28.81 |
| Time between diagnosis and blood draw | ≤ 3 months | 1062 (47.8) | 469.0 (426.1,516.1) | Ref. |
|  | >3 months | 1159 (52.2) | 520.0 (472.6,572.1) | 10.88* |
| **24-DHLan** |  |  |  |  |
| *Mutually adjusted for age, BMI, study region, diabetes, statin use, tumor stage, endocrine therapy* | | | | |
| Age at diagnosis (years) | < 55 | 172 (16.7) | 36.3 (32.5,40.6) | Ref. |
|  | 55 - 59 | 189 (18.4) | 38.2 (34.3,42.5) | 5.17 |
|  | 60 - 64 | 317 (30.9) | 37.9 (34.4,41.8) | 4.47 |
|  | 65 - 69 | 246 (24.0) | 36.5 (33.1,40.2) | 0.56 |
|  | 70 + | 103 (10.0) | 38.6 (34.0,43.8) | 6.41 |
| BMI (kg/m^2^) | < 18.5 | 20 (1.9) | 39.2 (30.5,50.4) | 4.54 |
|  | 18.5 -24.9 | 489 (47.6) | 37.5 (34.3,41.0) | Ref. |
|  | 25-29.9 | 369 (35.9) | 36.5 (33.4,39.9) | -2.65 |
|  | >30 | 149 (14.5) | 40.3 (36.1,44.9) | 7.42 |
| Study region | Hamburg | 489 (47.6) | 36.1 (33.0,39.4) | Ref. |
|  | Rhine-Neckar-Karlsruhe | 538 (52.4) | 38.7 (35.6,42.2) | 7.48* |
| Statin use | No | 637 (62.0) | 38.7 (35.5,42.2) | Ref. |
|  | Yes | 111 (10.8) | 33.1 (29.2,37.5) | -14.34 |
|  | Unknown | 277 (27.0) | 40.8 (37.4,44.5) | 5.44 |
| Stage | I | 514 (50.0) | 34.7 (32.0,37.6) | Ref. |
|  | IIa | 311 (30.3) | 36.0 (33.0,39.3) | 3.81 |
|  | IIb | 133 (13.0) | 36.5 (32.5,40.9) | 5.12 |
|  | IIIa | 69 (6.7) | 42.8 (37.0,49.6) | 23.52* |
| Endocrine therapy | None | 177 (17.2) | 38.7 (34.9,42.9) | Ref. |
|  | Tam + AI | 360 (35.1) | 36.5 (33.4,39.9) | -5.71 |
|  | Tam only | 347 (33.8) | 33.9 (31.1,37.0) | -12.37* |
|  | AI only | 109 (10.6) | 40.9 (36.3,46.1) | 5.66 |
|  | Unknown | 34 (3.3) | 37.2 (30.4,45.5) | -4 |
| Diabetes | No | 941 (91.6) | 42.1 (39.5,44.7) | Ref. |
|  | Yes | 83 (8.1) | 33.2 (29.2,37.8) | -21.00* |
| **25-HC** |  |  |  |  |
| *Mutually adjusted for stage, study region, time between diagnosis and blood collection* | | | | |
| Age at diagnosis (years) | < 55 | 332 (14.5) | 20.2 (18.9,21.6) | Ref. |
|  | 55 - 59 | 452 (19.8) | 21.0 (19.8,22.3) | 3.87 |
|  | 60 - 64 | 675 (29.6) | 20.2 (19.2,21.2) | -0.29 |
|  | 65 - 69 | 566 (24.8) | 20.3 (19.2,21.4) | 0.44 |
|  | 70 + | 257 (11.3) | 20.1 (18.6,21.7) | -0.76 |
| BMI (kg/m^2^) | < 18.5 | 1031 (45.2) | 20.0 (16.4,24.5) | -1.01 |
|  | 18.5 -24.9 | 35 (1.5) | 20.2 (19.4,21.2) | Ref. |
|  | 25-29.9 | 847 (37.1) | 20.0 (19.1,20.9) | -1.36 |
|  | >30 | 368 (16.1) | 21.8 (20.5,23.3) | 7.8* |
| Stage | I | 1137 (49.8) | 19.6 (18.9,20.3) | Ref. |
|  | IIa | 718 (31.5) | 20.1 (19.2,21.0) | 2.63 |
|  | IIb | 273 (12.0) | 20.3 (18.9,21.8) | 3.82 |
|  | IIIa | 154 (6.7) | 21.5 (19.6,23.7) | 10.07 |
| Study region | Hamburg | 1047 (45.9) | 24.5 (23.4,25.5) | Ref. |
|  | Rhine-Neckar-Karlsruhe | 1235 (54.1) | 16.9 (16.3,17.6) | -30.74** |
| Time between diagnosis and blood draw | ≤ 3 months | 1089 (47.7) | 21.6 (20.7,22.5) | Ref.. |
|  | >3 months | 1193 (52.3) | 19.2 (18.4,20.0) | -11.07** |
| **THC** |  |  |  |  |
| *Mutually adjusted for age, BMI, study region, parity, education, diabetes, hypertension, osteoporosis, aspirin use, tumor stage, tumor grade, endocrine therapy, chemotherapy, time between diagnosis and blood collection* | | | | |
| Age at diagnosis (years) | < 55 | 332 (14.5) | 3.9 (3.2,4.8) | Ref. |
|  | 55 - 59 | 452 (19.8) | 4.6 (3.8,5.6) | 17.08 |
|  | 60 - 64 | 675 (29.6) | 5.0 (4.2,6.0) | 27.54* |
|  | 65 - 69 | 566 (24.8) | 5.3 (4.4,6.3) | 34.04* |
|  | 70 + | 257 (11.3) | 5.3 (4.3,6.6) | 34.94 |
| BMI (kg/m^2^) | < 18.5 | 35 (1.5) | 5.1 (3.2,8.0) | 9.29 |
|  | 18.5 -24.9 | 1031 (45.2) | 4.6 (3.9,5.5) | Ref. |
|  | 25-29.9 | 848 (37.2) | 4.7 (4.0,5.6) | 1.64 |
|  | >30 | 368 (16.1) | 5.6 (4.6,6.8) | 19.77* |
| Study region | Hamburg | 1047 (45.9) | 8.6 (7.3,10.1) | Ref. |
|  | Rhine-Neckar-Karlsruhe | 1235 (54.1) | 2.7 (2.3,3.2) | -68.37** |
| Parity | 0 | 382 (16.7) | 4.8 (3.9,5.9) | Ref. |
|  | 1 | 643 (28.2) | 4.7 (3.9,5.7) | -0.64 |
|  | 2+ | 1257 (55.1) | 4.1 (3.5,4.9) | -13.31 |
| Education | Low | 1356 (59.4) | 4.2 (3.6,5.0) | Ref. |
|  | Medium | 598 (26.2) | 5.4 (4.5,6.6) | 27.70* |
|  | High | 328 (14.4) | 4.1 (3.3,5.1) | -4.17 |
| Aspirin | No | 1416 (62.1) | 5.1 (4.4,6.1) | Ref. |
|  | Yes | 265 (11.6) | 4.2 (3.4,5.2) | -17.85* |
|  | Unknown | 601 (26.3) | 5.2 (4.4,6.1) | 0.72 |
| Stage | I | 1137 (49.8) | 4.4 (3.8,5.2) | Ref. |
|  | IIa | 718 (31.5) | 4.8 (4.0,5.6) | 7.57 |
|  | IIb | 273 (12.0) | 5.1 (4.2,6.3) | 16.31 |
|  | IIIa | 154 (6.7) | 5.0 (3.9,6.5) | 14.08 |
| Endocrine therapy | None | 351 (15.4) | 5.1 (4.2,6.2) | Ref. |
|  | Tam + AI | 742 (32.5) | 4.4 (3.8,5.2) | -12.86 |
|  | Tam only | 855 (37.5) | 4.6 (4.0,5.5) | -8.61 |
|  | AI only | 255 (11.2) | 5.1 (4.2,6.3) | 0.50 |
|  | Unknown | 79 (3.5) | 4.9 (3.5,6.9) | -3.54 |
| Diabetes | No | 2070 (90.7) | 5.2 (4.6,6.0) | Ref. |
|  | Yes | 207 (9.1) | 4.5 (3.6,5.6) | -14.17 |
| Hypertension | No | 1338 (58.6) | 5.0 (4.2,5.9) | Ref. |
|  | Yes | 941 (41.2) | 4.7 (4.0,5.5) | -7.12 |
| Osteoporosis | No | 1986 (87.0) | 4.3 (3.8,5.0) | Ref. |
|  | Yes | 237 (10.4) | 5.4 (4.4,6.6) | 24.42* |
| Time between diagnosis and blood draw | ≤ 3 months | 1089 (47.7) | 5.1 (4.4,6.1) | Ref. |
|  | >3 months | 1193 (52.3) | 4.5 (3.9,5.3) | -11.81* |
| Chemotherapy | No | 1218 (53.4) | 4.4 (3.7,5.3) | Ref. |
|  | Yes | 1040 (45.6) | 5.3 (4.5,6.2) | 19.35* |
| **7a-HC** |  |  |  |  |
| *Mutually adjusted for age, BMI, study region, ERPR-status, endocrine therapy, time between diagnosis and blood collection* | | | | |
| Age at diagnosis (years) | < 55 | 330 (14.5) | 260.6 (243.8,278.6) | Ref. |
|  | 55 - 59 | 447 (19.7) | 277.6 (261.8,294.4) | 7.27 |
|  | 60 - 64 | 673 (29.6) | 278.3 (264.3,293.0) | 7.45 |
|  | 65 - 69 | 564 (24.8) | 282.6 (267.7,298.3) | 8.75* |
|  | 70 + | 257 (11.3) | 256.0 (237.6,275.9) | -1.55 |
| BMI (kg/m^2^) | < 18.5 | 34 (1.5) | 285.0 (235.4,345.0) | 11.53 |
|  | 18.5 -24.9 | 1027 (45.2) | 255.5 (244.5,267.0) | Ref. |
|  | 25-29.9 | 846 (37.3) | 281.2 (267.9,295.1) | 11.16** |
|  | >30 | 364 (16.0) | 312.4 (293.0,333.0) | 22.37** |
| Study region | Hamburg | 1036 (45.6) | 393.9 (376.6,412.1) | Ref. |
|  | Rhine-Neckar-Karlsruhe | 1235 (54.4) | 190.5 (182.6,198.6) | -53.04** |
| ER/PR | ER/PR + | 1575 (69.4) | 283.9 (272.0,296.4) | Ref. |
|  | ER+/PR- or ER-/PR+ | 374 (16.5) | 279.9 (262.8,298.2) | -1.50 |
|  | ER/PR- | 322 (14.2) | 258.6 (239.4,279.3) | -11.88* |
| Endocrine therapy | None | 347 (15.3) | 274.3 (256.3,293.6) | Ref. |
|  | Tam + AI | 740 (32.6) | 289.1 (274.3,304.7) | 12.38* |
|  | Tam only | 852 (37.5) | 289.3 (275.1,304.2) | 12.44* |
|  | AI only | 253 (11.1) | 254.3 (235.2,275.0) | -1.93 |
|  | Unknown | 79 (3.5) | 264.3 (233.1,299.6) | -1.93 |
| Time between diagnosis and blood draw | ≤ 3 months | 1084 (47.7) | 259.9 (248.7,271.5) | Ref. |
|  | >3 months | 1187 (52.3) | 288.7 (276.7,301.3) | 11.32** |
| **7b-HC** |  |  |  |  |
| *Mutually adjusted for age, BMI, study region, ERPR-status, endocrine therapy, chronic lung diseases, tumor grade* | | | | |
| Age at diagnosis (years) | < 55 | 330 (14.5) | 175.5 (155.7,198.0) | Ref. |
|  | 55 - 59 | 447 (19.7) | 189.4 (169.6,211.4) | 7.87 |
|  | 60 - 64 | 673 (29.6) | 193.4 (174.9,213.9) | 10.18 |
|  | 65 - 69 | 564 (24.8) | 198.0 (178.7,219.4) | 12.80 |
|  | 70 + | 257 (11.3) | 193.1 (169.0,220.6) | 10.01 |
| BMI (kg/m^2^) | < 18.5 | 1027 (45.2) | 230.5 (168.3,315.7) | Ref. |
|  | 18.5 -24.9 | 34 (1.5) | 178.0 (162.8,194.6) | 29.56 |
|  | 25-29.9 | 846 (37.3) | 193.2 (175.7,212.4) | 8.57 |
|  | >30 | 364 (16.0) | 219.1 (194.8,246.6) | 23.14* |
| Study region | Hamburg | 1036 (45.6) | 393.6 (359.8,430.6) | Ref. |
|  | Rhine-Neckar-Karlsruhe | 1235 (54.4) | 92.5 (84.7,100.9) | -76.51** |
| ER/PR | ER/PR + | 1575 (69.3) | 196.2 (179.8,214.0) | Ref. |
|  | ER+/PR- or ER-/PR+ | 375 (16.5) | 204.7 (182.3,229.8) | 4.33 |
|  | ER/PR- | 322 (14.2) | 172.9 (150.1,199.2) | -11.86 |
| Endocrine therapy | None | 347 (15.3) | 183.0 (161.9,206.9) | Ref. |
|  | Tam + AI | 740 (32.6) | 198.3 (179.1,219.5) | 8.35 |
|  | Tam only | 853 (37.5) | 205.5 (186.4,226.6) | 12.28 |
|  | AI only | 253 (11.1) | 170.9 (148.8,196.2) | -6.64 |
|  | Unknown | 79 (3.5) | 198.2 (160.3,245.1) | 8.28 |
| Lung disease | No | 2038 (89.7) | 177.1 (166.2,188.6) | Ref. |
|  | Yes | 234 (10.3) | 205.5 (180.8,233.7) | 16.09* |
| Grade | Low | 467 (20.6) | 194.5 (173.3,218.3) | Ref. |
|  | Medium | 1231 (54.2) | 191.3 (174.8,209.3) | -1.65 |
|  | High | 563 (24.8) | 186.6 (169.4,205.6) | -4.08 |
| **7-KC** |  |  |  |  |
| *Mutually adjusted for age, BMI, study region, ERPR-status and tumor grade* | | | | |
| Age at diagnosis (years) | < 55 | 332 (14.5) | 177.2 (161.2,194.8) | Ref. |
|  | 55 - 59 | 452 (19.8) | 207.4 (191.0,225.2) | 17.03* |
|  | 60 - 64 | 675 (29.6) | 197.6 (184.1,212.1) | 11.53 |
|  | 65 - 69 | 566 (24.8) | 205.9 (190.8,222.3) | 16.21* |
|  | 70 + | 257 (11.3) | 209.8 (188.3,233.8) | 18.4* |
| BMI (kg/m^2^) | < 18.5 | 35 (1.5) | 227.2 (172.0,300.1) | 26.00 |
|  | 18.5 -24.9 | 1031 (45.2) | 180.3 (170.1,191.1) | Ref. |
|  | 25-29.9 | 848 (37.2) | 203.2 (190.3,216.9) | 12.69* |
|  | >30 | 368 (16.1) | 251.8 (229.5,276.2) | 39.65** |
| Study region | Hamburg | 1047 (45.9) | 428.6 (404.3,454.3) | Ref. |
|  | Rhine-Neckar-Karlsruhe | 1235 (54.1) | 93.1 (87.9,98.6) | -78.27** |
| ER/PR | ER/PR + | 1583 (69.4) | 199.7 (187.8,212.4) | Ref. |
|  | ER+/PR- or ER-/PR+ | 376 (16.5) | 222.4 (198.0,249.8) | 11.36 |
|  | ER/PR- | 323 (14.2) | 171.7 (150.2,196.2) | -14.04* |
| Grade | Low | 469 (20.6) | 201.1 (184.4,219.3) | Ref. |
|  | Medium | 1234 (54.1) | 197.3 (185.9,209.3) | -1.89 |
|  | High | 568 (24.9) | 201.0 (187.3,215.8) | -0.01 |

Geometric means (95% CI) calculated using generalized linear models adjusted for age at diagnosis (continuous), BMI (continuous), and study region (Hamburg, Rhine-Neckar-Karlsruhe), additionally adjusted for exposures strongly associated with oxysterol concentrations in the primary analysis (p<0.05 and % difference ≥10%); except for the respective predictor variable.

*p-diff<0.05; **p-diff< 0.001

Abbreviations: CCI=Charlson Comorbidty Inde; CVD=cardiovascular diseases at baseline; statin and aspirin use reported at baseline; BMI=body mass index; parity=number of fullterm births; Endocrine therapy: None=neither SERM nor AI; Tam+AI=tamoxifen and aromatase inhibitors; Tam only=tamoxifen (no aromatase inhibitors); AI only = aromatase inhibitors (no tamoxifen): nM=nanomolar; LOD=level of detection; p-diff=percentage difference between extreme categories; 24-DHLan=24,25-dihydrolanosterol; 7-DC=7-dehydrocholesterol; 27-HC=27-hydroxycholesterol; 25-HC=25-hydroxycholesterol; 24S-HC=24S-hydroxycholesterol; 5a6a-EC=5α,6α-epoxycholesterol; 5b6b-EC=5β,6β-epoxycholesterol; THC=5α,6β-dihydroxycholestanol; 7a-HC=7α-hydroxycholesterol; 7b-HC=7β-hydroxycholesterol; 7-KC=7-ketocholesterol.

**References:**

1 Mutemberezi, V., Guillemot-Legris, O. & Muccioli, G. G. Oxysterols: From cholesterol metabolites to key mediators. *Progress in lipid research* **64**, 152-169, doi:10.1016/j.plipres.2016.09.002 (2016).

2 Gómez-Coronado, D., Lasunción, M. A., Martínez-Botas, J. & Fernández-Suárez, M. E. Role of cholesterol metabolism in the anticancer pharmacology of selective estrogen receptor modulators. *Seminars in Cancer Biology* **73**, 101-115, doi:<https://doi.org/10.1016/j.semcancer.2020.08.015> (2021).

3 Brown, A. J., Sharpe, L. J. & Rogers, M. J. Oxysterols: From physiological tuners to pharmacological opportunities. *British journal of pharmacology* **178**, 3089-3103, doi:10.1111/bph.15073 (2021).

4 Zmyslowski, A. & Szterk, A. Oxysterols as a biomarker in diseases. *Clin Chim Acta* **491**, 103-113, doi:10.1016/j.cca.2019.01.022 (2019).

5 Griffiths, W. J. & Wang, Y. Oxysterols as lipid mediators: Their biosynthetic genes, enzymes and metabolites. *Prostaglandins Other Lipid Mediat* **147**, 106381, doi:10.1016/j.prostaglandins.2019.106381 (2020).

6 Russell, D. W. Oxysterol biosynthetic enzymes. *Biochim Biophys Acta* **1529**, 126-135, doi:10.1016/s1388-1981(00)00142-6 (2000).

7 Decker, N. S. *et al.* Endogenous estrogen receptor modulating oxysterols and breast cancer prognosis: Results from the MARIE patient cohort. *British journal of cancer* **129**, 492-502, doi:10.1038/s41416-023-02315-w (2023).

8 Decker, N. S. *et al.* Circulating oxysterols and prognosis among women with a breast cancer diagnosis: results from the MARIE patient cohort. *BMC Medicine* **21**, 438, doi:10.1186/s12916-023-03152-7 (2023).
